# Psychological wellbeing and social emotional competence of Chinese children and adolescents in the post-pandemic era: patterns, determinants, and interrelations

**DOI:** 10.3389/fpubh.2025.1677632

**Published:** 2025-11-18

**Authors:** Gege Li, Tianjiao Chen, Feng Zhang, Peiyu Wang, Yan Yi, Xin Yin, Heng Luo

**Affiliations:** Faculty of Artificial Intelligence in Education, Central China Normal University, Wuhan, China

**Keywords:** psychological wellbeing, social emotional competence, children and adolescents, China, COVID-19, logistic regression analysis, qualitative analysis

## Abstract

**Background:**

The COVID-19 pandemic has presented distinct challenges to the psychological wellbeing (PWB) and social emotional competence (SEC) of Chinese children and adolescents due to prolonged prevention measures, but knowledge of their PWB and SEC status in the post-pandemic era—including distinctive patterns, essential determinants, and interrelationships—remains limited.

**Method:**

This study employed a self-report questionnaire with three sections (baseline characteristics, PWB scale, and SEC scale) to survey the PWB and SEC levels of Chinese children and adolescents in Gong'an County, central China. A total of 3,420 participants aged 8–16 from different areas in the district were surveyed and 2,848 valid responses were collected. Additionally, we identified 24 participants with low PWB and SEC scores and conducted follow-up interviews and family visits with them and their parents. Quantitative statistical analyses included descriptive analyses, analysis of variance (ANOVA), logistical regressions, and correlational analyses. Qualitative analyses were conducted to explain the statistical results as well as reveal emerging themed findings.

**Results:**

The quantitative data revealed moderate PWB and SEC levels among the participants, with significant variations based on baseline characteristics such as grade level, school location, academic rank, parent marital status, household economic condition, and left-behind status. Determinants influencing both PWB and SEC included academic rank, parent marital status, and household economic condition. The interrelationships between PWB and SEC dimensions also highlighted the importance of self-management for the wellbeing of Chinese youths. Furthermore, three theme findings were identified that are aligned with the quantitative results, focusing on PWB and SEC challenges, the impact of economic conditions on family dynamics, and the influence of grade obsession and self-esteem issues in the post-pandemic era.

**Conclusion:**

Teachers should monitor students' psychological and emotional wellbeing, especially those with poor academic performance, disadvantaged backgrounds, and unstable parental relationships. Non-left-behind children with high social awareness require equal attention. Effective interventions are needed to develop students' self-management and self-awareness, thus promoting their PWB.

## 1 Introduction

Psychological wellbeing (PWB) and social emotional competence (SEC) are fundamental to healthy development and good quality of life among children and adolescents during their transition into adulthood (1, 2). PWB is a core feature of mental health that encompasses multiple aspects of positive functioning and experiences such as hedonic and eudemonic happiness (3, 4). It has been linked with positive life outcomes such as healthy relationships, quality education, and gainful employment (5). SEC refers to a range of capabilities that allow people to regulate their emotions and interaction behaviors effectively at both the intra- and inter-personal levels (6–8). Longitudinal studies have associated children' SEC with higher school performance, work success, and mental health (9, 10), as well as decreased risky behaviors such as criminal activities and substance abuse (11).

Over the past 30 years, children and adolescents worldwide have been facing increasing challenges and threats to the development of their PWB and SEC owing to the complex interaction of social, political, economic, and cultural factors (12). Historically, up to 20% of children and adolescents worldwide suffered from a disabling mental health problem (e.g., depression and anxiety), and suicide ranked among the top five leading causes of death for adolescents (13). Similarly, the literature reveals the prevalence of SEC deficiency in childhood, as 5%−26% of the children have been estimated to suffer from social and emotional dysfunctions (14), which can lead to future problems such as maladjustment and peer victimization (15, 16). During the COVID-19 pandemic, the PWB and SEC of children and adolescents were further compromised due to issues such as prolonged social isolation, reduced physical activity, disrupted daily routines, and reduced access to professional support (17–19). Furthermore, compared to adults, the pandemic is believed to have caused persistent long-term adverse impacts on children and adolescents (20, 21).

Children and adolescents in China deserve special attention regarding their PWB and SEC conditions owing to two prominent characteristics of Chinese society. First, about 38% of rural youth in China have suffered long-term parental separation due to massive rural–urban migration since the 1980s, as rural laborers move to major cities for better job opportunities but leave their children behind (22, 23). Strong evidence has associated the left-behind experience with greater risk of mental and emotional problems such as social anxiety, emotional instability, low self-concept, and suicidal ideation (24–27). Second, China's educational and social ideologies place great emphasis on academic achievement, and admission to a good university is considered to be the best path toward individual success (28). Unfortunately, the school promotion rates are quite competitive in China (about 60% from middle school to regular high school and 27% to four-year colleges) (29), and failure in the promotion exams means the failure of family expectations and investment, which is known to trigger mental and emotional issues that can lead to adolescent suicidality (30, 31).

While serious concerns about the PWB and SEC of Chinese children and adolescents during the COVID-19 pandemic have attracted increasing research attention (2, 32–34), there are several limitations in the current literature. First, most studies were conducted early in the pandemic, which featured school closures, isolation, and quarantine; there is a dearth of research assessing the continued and cumulative impact of the pandemic on PWB and SEC in the post-pandemic era. Second, although the factors influencing PWB and SEC for children and adolescents have been studied, several social determinants essential to China's context lack systematic investigation, such as students' academic achievement, left-behind status, parent relationship, and school locations. Finally, despite the reciprocal relationship between PWB and SEC among children and adolescents, few studies have examined the two constructs concurrently in post-pandemic China, and thus, much remains unknown regarding the interrelations between PWB and SEC, as well as the similarity and difference in their distributions and influencing factors.

To address the current limitations in the literature, the present study examined the status of PWB and SEC among Chinese children and adolescents after 3 years of the COVID-19 pandemic, which featured frequent school closures and strict prevention measures. More importantly, we investigated several demographic and social determinants of PWB and SEC, with particular attention to the extent to which they can effectively predict psychological problems and social emotional dysfunctions in Chinese youth. We also compared the findings between PWB and SEC to explore their interrelations and similarities. The following questions guided our research inquiry:

(1) What are the PWB and SEC levels of children and adolescents in post-pandemic China? How do they vary across contextual and socio-demographic variables?
(2) Which contextual and socio-demographic factors serve as essential determinants of PWB and SEC for children and adolescents in post-pandemic China? How do they vary in their predictive capacities?
(3) What are the correlations and differences between the findings for PWB and SEC in the sampled population?

## 2 Literature review

### 2.1 PWB

Psychological wellbeing is defined as “the presence of the positive, which involves grappling with basic values and ideals of the human experience”, which includes aspects of self-acceptance, positive relations with others, autonomy, environmental mastery, purpose in life, and personal growth (3, 35). Ryff pointed out that there were two forms of success in PWB: bringing the person out of negative functioning, facilitating progression toward the restoration of positive functioning (36).

The present study used the scale measuring negative and positive functioning of adolescents, which includes five dimensions: positive affect (the experience of positive emotions and feelings), agitation (feelings or behaviors experienced when experiencing frustration or anger), depression (a mood disorder characterized by persistent feelings of sadness, hopelessness, and a lack of interest in activities), meaninglessness (a sense of lack of purpose or significance in life), and self-esteem (the overall evaluation of one's own worth or value) (37). Previous research studies indicated that PWB could be improved by behavioral interventions (38, 39). Positive PWB is associated with reduced depression symptoms, increased life satisfaction, positive interpersonal relationships, and life development (40, 41).

### 2.2 SEC

Social emotional competence is defined as “the process through which all young people and adults acquire and apply the knowledge, skills, and attitudes to develop healthy identities, manage emotions and achieve personal and collective goals, feel and show empathy for others, establish and maintain supportive relationships, and make responsible and caring decisions” (6). Elias et al. (42) defined SEC as “the ability to understand, manage, and express the social and emotional aspects of one's life in ways that enable the successful management of life tasks such as learning, forming relationships, solving everyday problems, and adapting to the complex demands of growth and development.”

The most widely used measurement of SEC consists of five core competencies: self-awareness (the capacity to understand personal emotions, thoughts, values, and contextual behavioral impacts), self-management (the ability to effectively manage emotions, thoughts, and behaviors across situations to reach goals), social awareness (the ability to understand and empathize with others from diverse backgrounds and contexts), relationship skills (the ability to establish and maintain healthy and supportive relationships, navigate settings with diverse individuals and groups), and responsible decision-making (the ability to make caring, constructive choices in diverse situations). Previous research studies have shown that social emotional learning programs can be effective in helping adolescents deal with stressors, reduce risk of developing mental health difficulties, foster positive outcomes, and promote asset development by enhancing cognitive, affective, and behavioral competences (43, 44).

### 2.3 Theoretical framework linking PWB and SEC

**Figure 1:**
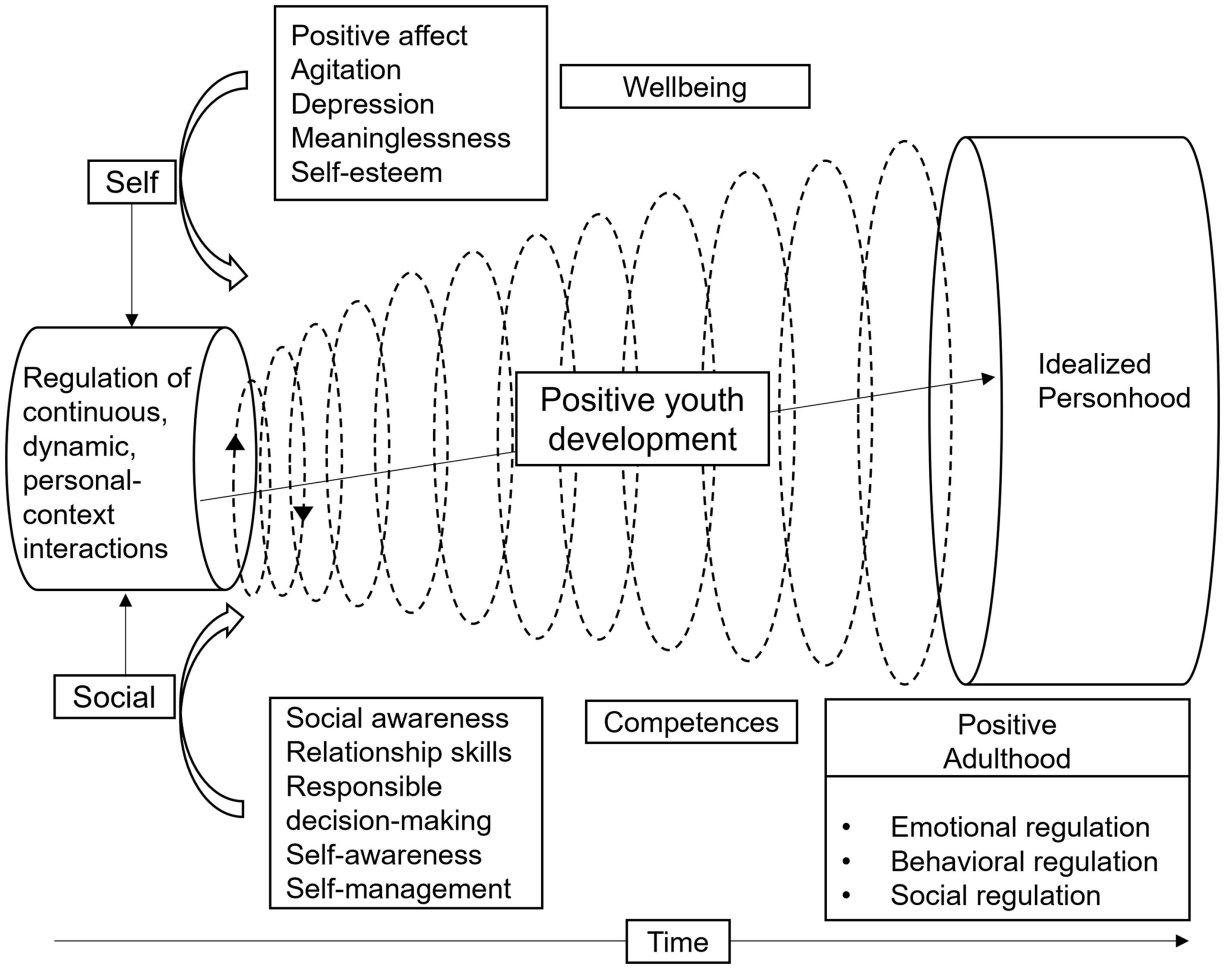
Theoretical framework based on PYD.

Figure 1 provides an illustration of the theoretical model employed in our research study, which was informed by the development contextual view of positive youth development (PYD) proposed by R. M. Lerner and Steinberg (45). PYD perspective focuses on the enhancement of adolescents' strengths, the facilitation of continuous and dynamic personal-context interactions, and the accentuation of the potential for development within positive environments (46). We consider that PWB and SEC are two crucial aspects for long-term development of adolescents. PWB focuses on the internal states and functions of the individual, whereas SEC centers on the competencies and adaptability demonstrated by individuals within social interactions.

There is a close link between PWB and SEC, with several researchers showing that higher SEC contributes to effective reduction of depression symptoms and positive development of PWB (47–49). Adolescents who demonstrate positive PWB and high levels of SEC often exhibit a more pronounced positive trend in their personal growth and development, which enables them to achieve their idealized personhood. Ultimately, these positive characteristics could lead to positive adulthood, which encompass sound emotional, behavioral, and social regulation skills (50, 51). Therefore, PYD serves as the ultimate goal for enhancing students' psychological wellbeing and social emotional competence.

### 2.4 Contextual and socio-demographic factors influencing PWB and SEC

Adolescents' positive youth development is shaped by the interplay of demographic, academic, and socio-economic factors. Among demographic factors, gender and grade are the most frequently examined. Previous research studies indicated that females were more likely to exhibit internalizing problems such as anxiety and depression, whereas males were more prone to externalizing behaviors such as aggression (52, 53). Adolescents in higher grade levels, confronted with intensified academic pressure and pubertal transitions, have been found to report increased depressive symptoms (54). Adolescence is a developmental stage often marked by challenges in emotional regulation due to heightened self-awareness and complex peer relations, which can significantly influence academic performance, interpersonal relationships, and overall PWB and SEC (55).

Regarding academic related factors, school context and academic performance are recognized as key determinants that may influence adolescents' PWB and SEC. Differences in school resources and teacher support, moderated by urban–rural context, contribute to variations in adolescents' PWB and SEC (56, 57). Meanwhile, within China's exam-oriented educational culture, academic performance significantly influences adolescents' PWB and SEC. Low-achieving students are more likely to experience psychological issues, such as anxiety, self-doubt, and undermined self-esteem (58, 59). Also, they tend to exhibit weaker social emotional skills, such as reduced motivation, impaired peer relationships, and limited self-regulation, which further constrain the development of SEC.

Socio-economic factors are also important. Research has shown that family cohesion and stable parental relationships serve as protective factors for adolescents' psychological adjustment, whereas parental marital conflict has been shown to predict adolescents' behavioral and psychological problems (60, 61). In addition, household economic conditions shape adolescents' access to essential living and educational resources, with economic hardship increasing the risk of mental health problems and limiting opportunities for social emotional development (62). Left-behind status increases vulnerability to emotional and behavioral problems, as the prolonged absence of parents often leads to disruptions in attachment, reduced emotional support, and fewer opportunities for socialization and guidance (63).

In summary, previous research studies have examined how demographic, academic, and socio-economic factors may influence adolescents' PWB and SEC. However, most studies have been limited to a single aspect and lack an integrated, multi-factor perspective. Therefore, the present study incorporates eight contextual and socio-demographic variables, offering a more comprehensive interpretation of the factors influencing children and adolescents' PWB and SEC.

## 3 Materials and methods

### 3.1 Research design

We conducted a cross-sectional design to explore the influencing factors and relationship between PWB and SEC. A cross-sectional design is mainly focus on collecting, analyzing data from a large population at a specific time period among the targeted population (64). Therefore, a cross-sectional study allows the investigation of relationships among various variables. In our study, the predicting factors are demographic variables, including school location, academic rank, left-behind status, parent marital status, household economic condition, and only child status.

### 3.2 Research context, sampling, and procedures

To assess the status of PWB and SEC of Chinese children and adolescents, we selected the county of Gong'an, which is located in central China, as the site of investigation for two reasons. First, Gong'an is a typical county in Hubei province, which features a mixed socioeconomic structure and above-average education quality. The primary industry of Gong'an is agriculture (e.g., rice and grape cultivation). Like many other counties in China, residents in Gong'an routinely migrate to major cities for better work opportunities, which has resulted in a high proportion of left-behind children in the area.

A total of 3,420 students from the target population (age 8–16 years) participated in this study. They were from four elementary schools and two middle schools located in three different district area types that vary by economic development status: village, township, and city. We used a stratified random sampling technique to first divide the population by school and grade to create strata and then randomly selected 50%−90% of the classes in research sample.

The investigation process comprised three stages. In the first stage (February 28–March 3), we visited the school sites to administer the survey. We used paper-based questionnaires to collect responses from the participants, because they were prohibited from bringing their mobile phones to school. In the second stage (April 10–June 15), we screened the survey data to exclude invalid questionnaire responses. The exclusion criteria were as follows: (1) key information such as gender or grade was missing; and (2) all questionnaire items received the same rating. A total of 2,848 questionnaires were deemed valid, for a valid response rate of 83.3%. In the third stage (June 30–July 3), we further identified 23 participants with low PWB and SEC survey scores and conducted in-depth follow-up interviews with them and their parents during family visits.

### 3.3 Sample size calculation

The sample size calculation was conducted using G^*^ Power (version 3.1.9.4). We used the Z test to estimate the required minimum sample size for the logistic regression analysis used in this study. The calculation parameters were set as follows: the odds ratio was set at 1.5, Pr(*Y* = 1|*X* = 1) H0 at 0.5, the α error at 0.05, and the confidence interval at 95% (two-sided test). The final calculated minimum sample size was 337. The sample size in this study (*N* = 2,848) is larger than the calculated result and thus can yield sufficient power to identify significant determinants.

### 3.4 Ethical considerations

The research protocol was approved by the Institutional Review Board of Central China Normal University (CCNU-IRB-202306002a) and was then reviewed and endorsed by the local school administrators and a senior psychotherapist. The informed consent forms were distributed to both the students and their parents or legal guardians 3 days before the survey administration with the assistance of the head teachers. Students were required to submit both their own written consent and parental consent forms before filling out the survey. During our visits to the school sites and selected families, the students and their parents were once again informed of the study purpose, their right to anonymity, and the opt-out options.

### 3.5 Data collection instruments

The measurement instrument in this study was a self-reported questionnaire that had three main sections: basic information, PWB scale, and SEC scale. Detailed items are provided in Supplementary material Data sheet 1.

#### 3.5.1 Basic information

This section consisted of 13 items, which collected information about the participants' baseline characteristics, including gender, grade, school location, academic rank, parent marital status, household economic condition, only child status, and left-behind status. This basic information was considered to be important determinants of PWB and SEC.

#### 3.5.2 PWB scale

The structure and items were informed by Umberson and Gove (37). We made minor revisions to the scale to improve students' understanding of the item descriptions by removing double negatives and changing second-person pronouns to first-person pronouns. The PWB scale was divided into five subscales with 24 items: positive affect (4 items), agitation (5 items), depression (5 items), meaninglessness (5 items), and self-esteem (5 items). It is worth noting that the item ratings for the subscales for agitation, depression, and meaninglessness were reversed before computing the PWB total scores and sub-scores so that high scores represent a high level of PWB. All items were rated on a 5-point Likert scale ranging from 1 (strongly disagree) to 5 (strongly agree).

#### 3.5.3 SEC scale

The measurement of students' SEC with 25 items was evenly distributed into five subscales (5 items each): self-awareness, self-management, social awareness, relationship skills, and responsible decision-making. The measurement items of the SEC scale were based on the questionnaire created by Zhou and Ee (65), and there were no modifications of the measuring items. The dimension of SEC was informed by the CASEL framework (6). All items were rated on a 5-point Likert scale ranging from 1 (strongly disagree) to 5 (strongly agree).

Preliminary analysis was conducted to evaluate the reliability and validity of the PWB and SEC scale, and the key results are presented in Table 1. The scale reliability was calculated using Cronbach's α in SPSS, with an α value greater than 0.7 indicating good internal reliability. The scale validity was determined by both convergent and discriminant validity (66). Acceptable convergent validity requires factor loading values greater than 0.4, composite reliability (CR) greater than 0.6, and average variance extracted (AVE) values greater than 0.5 (67). Discriminant validity requires that square root of the AVE (√AVE) is greater than the correlation coefficient between constructs (68). We conducted confirmatory factor analysis (CFA) in AMOS to calculate factor loadings. The coefficient of factor loading reflects the degree of association between variables and factors. As shown in Table 1, most statistical assumptions for Cronbach's α, factor loading values, CR, AVE, and √AVE are met, which suggests an overall good reliability and validity of the measurement.

**Table 1:** Key statistics for reliability and validity tests.

**Constructs**	**Items**	**α**	**Factor loadings**	**CR**	**AVE**	**√AVE**
Baseline characteristics	13	NA	NA	NA	NA	NA
Psychological wellbeing	25	0.916				
Positive affect	4	0.704	[0.476–0.804]	0.732	0.417	0.646
Agitation scale	5	0.748	[0.450–0.729]	0.748	0.377	0.614
Depression	5	0.843	[0.661–0.776]	0.845	0.522	0.722
Meaninglessness	5	0.904	[0.671–0.872]	0.906	0.659	0.812
Self-esteem	5	0.782	[0.564–0.729]	0.781	0.419	0.647
Social emotional competence	25	0.896				
Self-awareness	5	0.736	[0.417–0.715]	0.758	0.391	0.625
Self-management	5	0.842	[0.563–0.794]	0.847	0.530	0.728
Social awareness	5	0.801	[0.558–0.748]	0.804	0.453	0.673
Relationship skills	5	0.678	[0.343–0.679]	0.709	0.338	0.581
Responsible decision-making	5	0.818	[0.638–0.753]	0.820	0.478	0.691

NA, not applicable; CR, composite reliability; AVE, average variance extracted; √AVE, square root of the AVE.

### 3.6 Contextual and socio-demographic factors

In this study, we included eight contextual and socio-demographic variables and classified them into three categories: demographic, academic-related, and socio-economic. The rationale for selecting these variables is grounded in previous findings on the determinants of adolescents' PWB and SEC, and is further informed by the unique educational and societal conditions that characterize China in the post-pandemic. The demographic variables (gender, grade level, and only child status) capture the fundamental developmental characteristics of adolescents. PWB and SEC may vary across gender and grade level, reflecting both biological transitions and developmental challenges during adolescence. The only child status, rooted in China's one-child policy, is associated with resource allocation and parental expectations.

The academic-related variables (school location and academic rank) reflect the critical role of school contexts in shaping adolescents' development. School location captures disparities in educational resources and social support across different regional settings. Academic rank is particularly salient in China's exam-oriented educational culture, as it not only shapes students' self-concept but also exerts profound and lasting effects on their PWB and SEC.

The socio-economic variables (parent marital status, household economic condition, and left-behind status) were included to reflect the influence of family and socio-economic circumstances on adolescents' development. Parent marital status indicates family stability, with marital disruption often linked to greater risks of psychological distress. Household economic condition shapes access to economic and educational resources. The left-behind status, a phenomenon unique to China resulting from large-scale rural–urban labor migration, which has been associated in prior studies with psychological distress and difficulties in SEC. Overall, these variables provide a contextual foundation for examining the determinants of adolescents' PWB and SEC.

### 3.7 Analytical techniques

We used three major statistical approaches to analyze the quantitative data in this study. First, we employed descriptive analyses and a series of one-way analysis of variance (ANOVA) to examine patterns of PWB and SEC for Chinese children and adolescents and any differences linked to baseline characteristics. Although the Likert-scale survey data do not meet the assumption of normal distribution, we consider ANOVA is appropriate for this study, since ANOVA exhibits robustness against deviations from normality and heterogeneity of variances, particularly for large sample size (69). Second, we conducted multiple logistic regressions to identify and compare the essential determinants that significantly predict PWB and SEC in the post-pandemic era. Multiple logistic regression is suitable for regression analysis when the dependent variable is categorical and comprises more than three categories. In this study, PWB and SEC are clustered into three categories of high, medium, and low, serving as the dependent variables for prediction. Third, we performed correlational analyses to explore the correlation between PWB and SEC and interrelations among their key constructs.

We also analyzed the fieldnote and interview data qualitatively to formulate themed findings to assist in the meaningful interpretation of the quantitative results. Based on the questionnaire results, we selected 23 students with lower PWB and SEC scores for field visits, obtaining a total of 44,379 Chinese characters of field logs and interview records. During the field visits, a four-member research team—including two teachers and two graduate researchers—was divided into two groups. One group conducted semi-structured interviews with parents, while the other interviewed students to prevent students from feeling constrained when expressing their views in front of their parents. To protect participants' privacy, no audio recordings were made. Each interview was conducted by two researchers—one leading the conversation and the other taking detailed real-time notes, which were immediately cross-checked for accuracy. Both students' and parents' interviews were analyzed together, with students' narratives serving as the primary data source and parents' accounts providing supplementary contextual insights. The interviews were organized around a set of topics, including family and socio-economic background, parent–child communication and emotional support, academic performance and learning habits, individual traits and personality development, and the perceived impact of the COVID-19 pandemic on students' learning, emotions, and family dynamics. Researchers also took detailed field notes, documenting family environments, interpersonal interactions, and non-verbal behaviors, which were later incorporated into the qualitative analysis.

Subsequently, we analyzed the qualitative data followed by the thematic analysis procedures proposed by Braun and Clarke (70). First, we familiarized ourselves with data by reading through the field notes and transcripts before coding. Then, we generated the initial codes by using the four coding techniques specified in the coding manual by Saldaña (71): (1) structural coding based on our research questions and measuring constructs; (2) *in vivo* coding that captures the vividness and authenticity of the commentary; (3) vs. coding that captures complexity and diversity of influencing factors. Finally, we identified six main themes through the analysis, including psychological wellbeing, social emotional competence, socio-economic conditions, family factors, individual factors, and academic factors. Completed codes see in Supplementary material Data sheet 3. These categories were later synthesized into three theme findings to provide a more coherent interpretation of the qualitative findings. All original interview transcripts have been translated into English and were provided in Supplementary material Data sheet 4.

## 4 Results

### 4.1 PWB and SEC levels among baseline characteristics

The mean PWB and SEC scores for the participants were 3.635 and 3.796, respectively. Among the PWB subscale, positive affect received the most favorable ratings (*M* = 3.948), while depression received the lowest ratings (*M* = 3.272). Among the SEC subscales, self-awareness received the highest ratings (*M* = 4.123), while self-management received the lowest ratings (*M* = 3.492). The ANOVA results revealed significant differences in participants' PWB and SEC scores for various baseline characteristics, as shown in Table 2. All baseline characteristics, except for only child status, had a significant impact on participants' PWB. Six characteristics (grade level, school location, academic rank, parental marital status, household economic condition, and left-behind status) also led to different SEC levels among the participants. Similar to PWB, participants' SEC levels were not influenced by whether they were the only child in the family or not. The patterns in which the baseline characteristics affected participants' PWB and SEC were generally consistent, with the only difference being the variable of gender, which caused a difference in PWB but not in SEC.

**Table 2:** Differences in psychological wellbeing (PWB) and social emotional competence (SEC) scores by baseline characteristics.

**Variables**	**PWB_M(SD)_**	***p***	**SEC_M(SD)_**	***p***
**Gender**		**0.016**		0.863
Male (*n =* 1,371)	3.669 (0.718)		3.794 (0.597)	
Female (*n =* 1,477)	3.603 (0.752)		3.797 (0.557)	
**Grade level**		**<0.001**		**<0.001**
3 (*n =* 294)	3.746 (0.612)		3.839 (0.579)	
4 (*n =* 301)	3.702 (0.704)		3.866 (0.572)	
5 (*n =* 393)	3.788 (0.664)		3.850 (0.541)	
6 (*n =* 277)	3.658 (0.763)		3.810 (0.590)	
7 (*n =* 685)	3.723 (0.763)		3.827 (0.577)	
8 (*n =* 616)	3.412 (0.774)		3.703 (0.583)	
9 (*n =* 282)	3.481 (0.680)		3.712 (0.565)	
**School location**		**<0.001**		**<0.001**
Village (*n =* 229)	3.596 (0.606)		3.710 (0.513)	
Township (*n =* 747)	3.423 (0.714)		3.629 (0.575)	
County (*n =* 1,872)	3.724 (0.743)		3.873 (0.569)	
**Academic rank (self-report)**		**<0.001**		**<0.001**
Poor (*n =* 151)	3.109 (0.800)		3.287 (0.711)	
Below average (*n =* 355)	3.285 (0.728)		3.588 (0.553)	
[-0.5pt] Above average (*n =* 1,996)	3.671 (0.694)		3.815 (0.542)	
Excellent (*n =* 346)	4.012 (0.689)		4.120 (0.492)	
**Left-behind status**		**<0.001**		**<0.001**
Father employed (*n =* 746)	3.671 (0.757)		3.825 (0.600)	
Mother employed (*n =* 120)	3.674 (0.704)		3.846 (0.629)	
Double-left-behind (*n =* 799)	3.539 (0.714)		3.710 (0.554)	
Non-left-behind (*n =* 1,025)	3.736 (0.716)		3.855 (0.558)	
Other (*n =* 47)	3.353 (0.726)		3.660 (0.562)	
Not reported (*n =* 111)	3.218 (0.748)		3.665 (0.584)	
**Parent marital status**		**<0.001**		**<0.001**
Divorced (*n =* 334)	3.394 (0.767)		3.663 (0.567)	
Married (*n =* 2,364)	3.693 (0.718)		3.824 (0.572)	
Other (*n =* 150)	3.251 (0.745)		3.639 (0.597)	
**Household economic condition**		**<0.001**		**<0.001**
Low (*n =* 163)	3.352 (0.749)		3.530 (0.562)	
Medium (*n =* 1,880)	3.592 (0.720)		3.741 (0.572)	
High (*n =* 805)	3.791 (0.744)		3.977 (0.540)	
Only child status		0.229		0.171
Yes (*n =* 992)	3.657 (0.757)		3.816 (0.574)	
No (*n =* 1,856)	3.622 (0.726)		3.785 (0.577)	

M, mean; SD, standard deviation; Double-left-behind refers to children and adolescents whose parents are both migrant workers away from home. Bold values indicate statistically significant results: *p* < 0.05; *p* < 0.01; *p* < 0.001.

**Figure 2:**
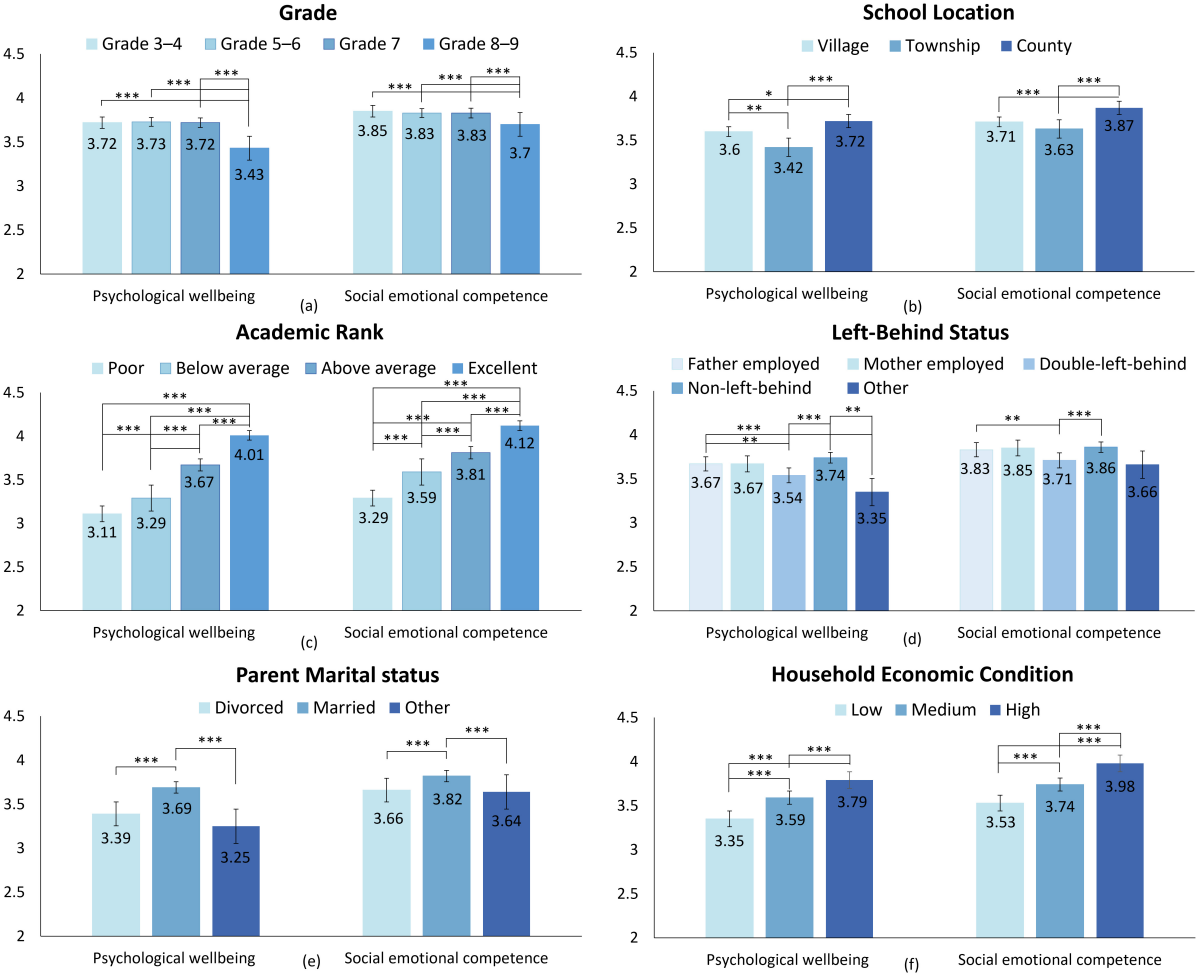
Differences in psychological wellbeing (PWB) and social emotional competence (SEC) among various baseline characteristics: **(a)** PWB and SEC differences among different grades; **(b)** PWB and SEC differences across school locations; **(c)** PWB and SEC differences across academic ranks; **(d)** PWB and SEC differences across types of left-behind status; **(e)** PWB and SEC differences among different parental marital statuses; **(f)** PWB and SEC differences across household economic conditions. *Post hoc* analysis: *p < 0.05; **p < 0.01; ***p < 0.001. “Double-left-behind” refers to children and adolescents whose parents are both migrant workers away from home.

To further compare participants' PWB and SEC levels against their baseline characteristics, we plotted a series of bar charts to demonstrate the between-group variance; significant differences are marked with asterisk(s), as shown in Figure 2. The baseline characteristics can be classified into two categories: school-related characteristics including grade, school location, and self-reported academic rank; and family-related characteristics, including left-behind status, parent marital status, and household economic condition. Overall, the PWB and SEC scores demonstrate similar patterns across different baseline characteristics.

Regarding school-related characteristics, Figure 2a shows that participants from Grades 8–9 reported the lowest PWB and SEC scores, while no significant differences were identified for the other grade levels. Figure 2b reveals that the participants attending city schools were associated with the highest PWB and SEC scores. Participants from village schools outscored those from township schools in PWB, but no such difference was observed in SEC scores. Figure 2c suggests a mapping relationship between participants' self-reported academic ranks and their PWB and SEC scores, with high-achieving students associated with higher PWB and SEC levels.

Regarding family-related characteristics, Figure 2d reveals that both the PWB and SEC scores of the participants declined as their left-behind status worsened. The participants living with both parents at home reported the highest scores, followed by those living with only one parent and those with neither parent at home. The participants from the “other” group, which involved complex situations such as the death or imprisonment of a parent, scored the lowest. Figure 2e shows that the marital status of parents had a significant influence on participants' PWB and SEC scores: divorced and other (e.g., separation, alienated) were associated with lower scores. Figure 2f suggests a positive correlation between participants' household economic condition and their PWB and SEC: the participants with limited access to resources (e.g., a private room, study desk, and personal computer) tended to report lower scores.

### 4.2 Essential determinants of PWB and SEC

We employed K-means cluster analysis to divide the participants into three groups (low, medium, and high) using the five subscale scores of PWB and SEC, respectively. Participants were divided into three cluster groups according to a low (*n* = 637), medium (*n* = 1,152), and high level of PWB (*n* = 1,059), and three cluster groups based on a low (*n* = 528), medium (*n* = 1,371), and high level of SEC (*n* = 949). In the subsequent logistic regressions, high-level groups of PWB and SEC were set as reference category. We focused on comparing the SEC and PWB scores between the high and low groups because such comparisons enable the identification of key factors influencing PWB and SEC. It is important to note that the average scores of the low-level groups were below the midpoint of the 5-point Likert scale, which indicates the prevalence of PWB and SEC issues within these groups. Additionally, the participants with a medium level of PWB and SEC account for the largest proportion of the sampled population.

Multiple logistic regressions were conducted to determine whether baseline characteristics can be considered as essential determinants of PWB and SEC. The assumptions of observations being independent and independent variables being linearly related to the log were checked and met. When all baseline characteristics are considered together, they can significantly predict whether or not a participant belongs to the low-level groups for PWB (χ^2^ = 380.831, *p* < 0.001, d*f* = 36) and SEC (χ^2^ = 353.717, *p* < 0.001, d*f* = 36). It is worth noting that the baseline characteristics that significantly predict low-level PWB and SEC are mostly identical to those predicting medium-level PWB and SEC, respectively. Consequently, we present only the regression results for low-level PWB and SEC in the main text and include the complete results in Supplementary material Data sheet 2.

Table 3 presents the odds ratios for each characteristic condition in the model predicting low-level PWB. The results indicate that gender, academic rank, school location, grade level, parent marital status, and household economic condition are significant determinants of PWB. Low-achieving girls in higher grade levels from township schools are at greater risk of experiencing psychological issues. Moreover, the predicaments in families such as deteriorated marital status and poor economic condition also increase the likelihood of PWB problems. However, whether a participant is the only child in family or has been left behind appears to have only a limited impact on PWB.

**Table 3:** Key regression results for predicting psychological wellbeing (PWB).

**Variables**	**B**	**SE**	**Wald**	***p***	**Exp(B)**	**95% CI**	
						**Lower**	**Upper**
Intercept	−0.262	0.52	5.882	0.015			
**Gender**							
Male	−0.420	0.112	14.141	**<0.001**	0.657	0.528	0.818
Female	0^b^						
**Grade**							
3–4	−0.683	0.185	13.613	**<0.001**	0.505	0.351	0.726
5–6	−0.597	0.176	11.55	0.001	0.55	0.39	0.777
7	−0.784	0.151	27.073	**<0.001**	0.456	0.34	0.613
8–9	0^b^						
**School location**							
Village	−0.044	0.24	0.033	0.856	0.957	0.598	1.533
Township	0.359	0.154	5.434	0.02	1.432	1.059	1.937
County	0^b^						
**Academic rank**							
Poor	2.604	0.327	63.579	**<0.001**	13.516	7.127	25.634
Below average	1.918	0.257	55.873	**<0.001**	6.808	4.117	11.258
Above average	0.964	0.205	22.118	**<0.001**	2.621	1.754	3.916
Excellent	0^b^						
**Left-behind status**							
Father employed	−0.120	0.462	0.068	0.794	0.887	0.359	2.192
Mother employed	−0.562	0.516	1.185	0.276	0.57	0.207	1.568
Double-left-behind	−0.128	0.457	0.078	0.78	0.88	0.359	2.154
Non-left behind	−0.390	0.462	0.71	0.399	0.677	0.274	1.676
Other	0^b^						
**Parent marital status**							
Other	1.183	0.257	21.172	**<0.001**	3.264	1.972	5.402
Divorced	0.613	0.182	11.29	0.001	1.846	1.291	2.64
Married	0^b^						
**Household economic condition**							
Low	1.096	0.265	17.168	**<0.001**	2.993	1.782	5.027
Medium	0.433	0.126	11.835	0.001	1.542	1.205	1.975
High	0^b^						
**Only child status**							
Yes	−0.089	0.116	0.596	0.44	0.914	0.729	1.148
No	0^b^						

SE, standard error; CI, confidence interval; Double-left-behind refers to children and adolescents whose parents are both migrant workers away from home, and b refers to the baseline standard used for comparison with other variables. Bold values indicate statistically significant results: *p* < 0.05; *p* < 0.01; *p* < 0.001.

Table 4 presents the odds ratios for each baseline characteristic in the model predicting low-level SEC. A similar pattern was found: school location, academic rank, parental marital status, and household economic condition were significant predictors, whereas only child and left-behind status exerted minimal influence. Notably, the significant predictors of low SEC mirrored those for PWB, underlying the intertwined nature of these outcomes. Specifically, academic rank, school location, and family-related factors consistently emerged as significant determinants across both regression models.

**Table 4:** Key regression results for predicting social emotional competence (SEC).

**Variables**	***B***	**SE**	**Wald**	***p***	**Exp(B)**	**95% CI**	
						**Lower**	**Upper**
Intercept	0.853	0.413	4.277	0.039			
**Gender**							
Male	0.128	0.089	2.072	0.15	1.137	0.955	1.354
Female	0^b^						
**Grade**							
3–4	0.172	0.144	1.415	0.234	1.187	0.895	1.576
5–6	0.08	0.142	0.315	0.575	1.083	0.82	1.43
7	0.106	0.125	0.717	0.397	1.112	0.87	1.421
8–9	0^b^						
**School location**							
Village	−0.119	0.184	0.416	0.519	0.888	0.619	1.274
Township	−0.303	0.132	5.275	**0.022**	0.739	0.57	0.957
County	0^b^						
**Academic rank**							
Poor	−1.500	0.31	23.415	**<0.001**	0.223	0.122	0.41
Below average	−1.226	0.195	39.362	**<0.001**	0.293	0.2	0.43
Above average	−0.677	0.128	27.788	**<0.001**	0.508	0.395	0.653
Excellent	0^b^						
**Left-behind status**							
Father employed	−0.134	0.378	0.125	0.724	0.875	0.417	1.834
Mother employed	0.155	0.42	0.136	0.713	1.167	0.513	2.656
Double-left-behind	−0.345	0.374	0.85	0.356	0.708	0.34	1.475
Non-left behind	−0.218	0.377	0.335	0.563	0.804	0.384	1.684
Other	0^b^						
**Parent marital status**							
Other	−0.264	0.231	1.306	0.253	0.768	0.488	1.208
Divorced	−0.335	0.164	4.173	**0.041**	0.715	0.519	0.986
Married	0^b^						
**Household economic condition**							
Low	−0.776	0.233	11.054	**0.001**	0.46	0.291	0.727
Medium	−0.525	0.097	29.27	**<0.001**	0.592	0.489	0.716
High	0^b^						
**Only child status**							
Yes	−0.006	0.093	0.004	0.948	0.994	0.828	1.194
No	0^b^						

SE, standard error; CI, confidence interval; Double-left-behind refers to children and adolescents whose parents are both migrant workers away from home, and b refers to the baseline standard used for comparison with other variables. Bold values indicate statistically significant results: *p* < 0.05; *p* < 0.01; *p* < 0.001.

### 4.3 Correlation analysis among the key constructs of PWB and SEC

**Figure 3:**
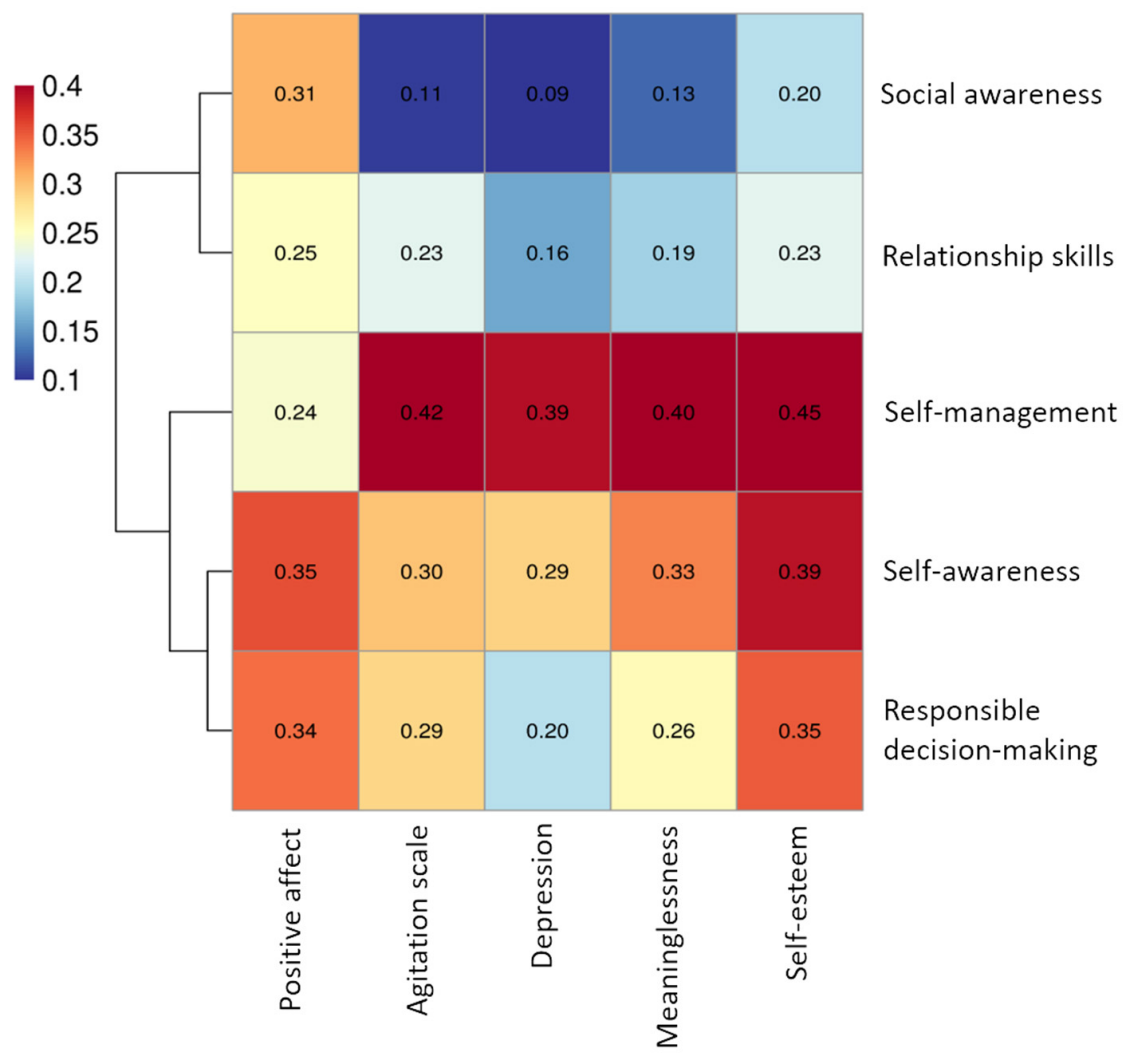
Correlations among the key constructs.

Correlation analysis was conducted to further examine the relationship among the key constructs of PWB and SEC. As shown in Figure 3, there is a moderately low correlation between various dimensions of PWB and SEC. Self-management shows the strongest correlation with participants' self-esteem (*r* = 0.45) and also exhibits a moderate correlation with meaninglessness, depression, and agitation. Conversely, the results indicate that participants' social awareness exhibits the weakest correlation with PWB, particularly with depression (*r* = 0.09).

### 4.4 Themed findings

**Figure 4:**
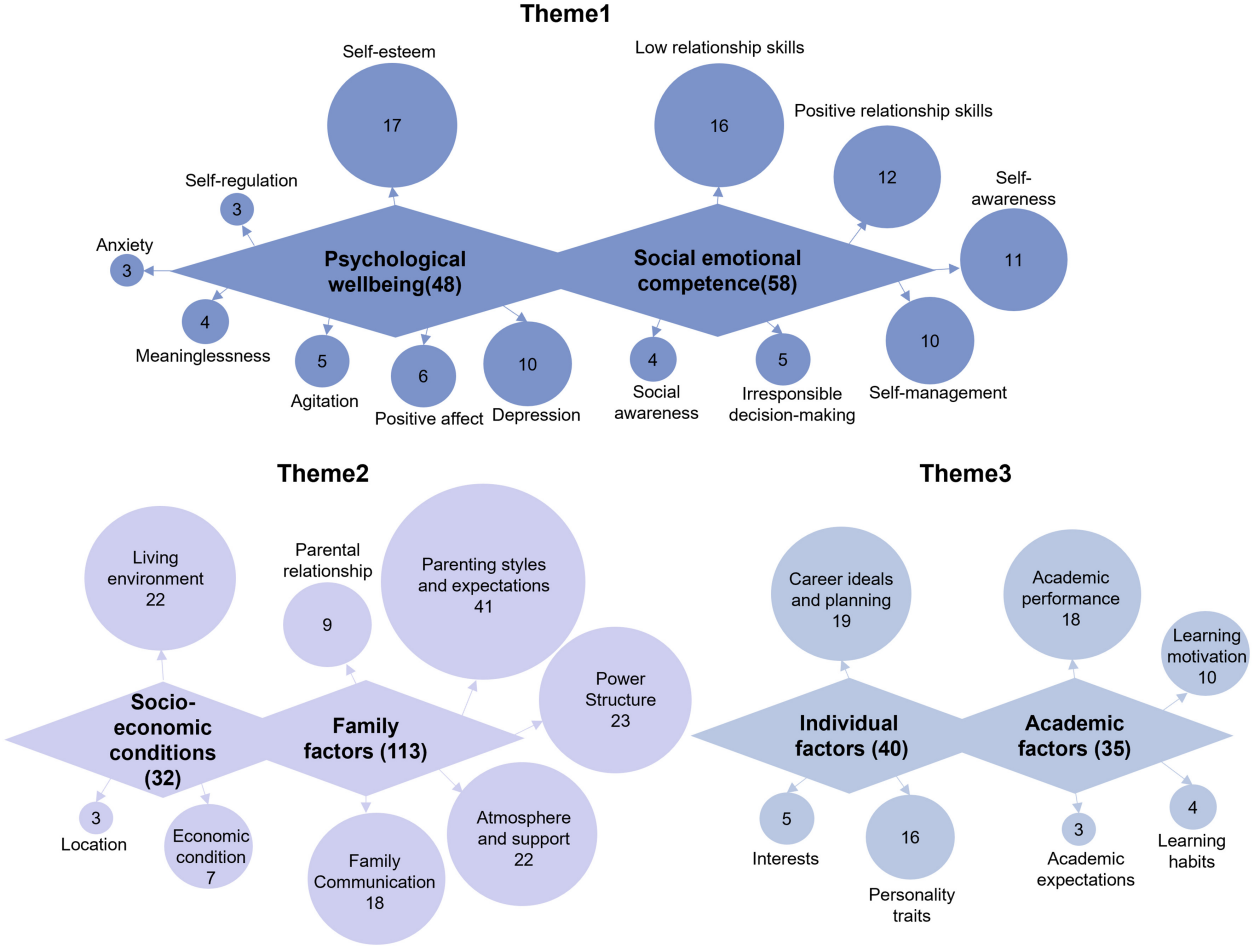
Themed findings of the qualitative results.

As shown in Figure 4, the six coded main themes—psychological wellbeing, social-emotional competence, socio-economic conditions, family factors, individual factors, and academic factors—were visually grouped into three color clusters, each corresponding to one theme finding (Theme 1–3). This visualization demonstrates how related domains were synthesized into broader interpretive narratives.

Theme 1: Students face more severe PWB issues than SEC challenges in the post-pandemic era.

Although the quantitative results reveal a slight decrement in students' PWB scores compared to SEC scores, our qualitative analysis implies a greater prevalence of PWB issues than SEC challenges. The learning losses arising from school closure and disruption was frequently mentioned during the interview, exacerbating student anxiety and stress. Several students told us that their academic performance was “quite alright” previously but the intermittent online studying during the pandemic had “ruined that”. This decline often led to reprimands from parents and teachers, inducing a sense of estrangement and disheartenment. A seventh-grade girl even confessed her suicidal ideation, feeling the learning loss during pandemic had dashed her hopes of entering high school, leaving her life meaningless. Moreover, post-pandemic schools adopted stricter measures such as increased study hours and workloads to recoup learning losses, which were associated by many students with symptoms of academic aversion, chronic fatigue, and sleep disturbance—all stressors contributing to mental health problems.

Contrary to the widespread pandemic concerns, students' social and emotional development proved to be more resilient than expected. Students were proficient in leveraging digital apps (e.g., TikTok) and online platforms (e.g., Eggy Island) to maintain social connections during and after the pandemic, showing a remarkable ability of Generation Alpha to adapt to changing circumstances. Several students attributed their personal growth to their online social experiences, citing benefits such as wider social circle, improved confidence, and developed empathy. For instance, a sixth-grader aspired to become an internet celebrity, emphasizing the need for developing eloquence, observance, and personal charm. However, we found social impressions can also be misleading: external factors (e.g., family upbringing, societal expectation) shaping social awareness and interpersonal skills do not necessarily foster concurrent PWB development. In fact, children from single-parent or divorced families tend to be more obedient, understanding, and adept at reading people's emotions, but they often find themselves trapped in negative emotions such as loneliness and helplessness.

Theme 2: Safe haven or stress hotbed? Implicit consequences of economic conditions on family dynamics.

The influence of the original family on psychological health and socio-emotional development is profound and multifaceted. During the interviews, nearly all students with low PWB and SEC scores expressed dissatisfaction with their family dynamics. Common complaints included difficulties in communication, a lack of understanding and emotional support, tense family relationship, poor parental modeling, and excessive control. For them, the family is no longer a shelter from worry and stress but a constant source of them. For example, a sixth-grade boy expressed fear of his father after witnessing him smash the television set in a fit of rage due to the boy's watching TV. Additionally, parents' unrealistic expectations often impose great mental stress on children. “The thought of becoming a disappointment to my family in 2 years (high school entrance exam) makes me feel numb in my limbs and unable to sleep”, a seventh-grade girl confessed. Lastly, children were sensitive to parental estrangement. Many have mentally prepared themselves for the forthcoming divorce, but were not ready for accepting step-parents, leading to long-term melancholy and gradual social withdrawal.

Through observations and interviews, it has become evident that economic pressures compelled families to prioritize financial gains over family stability, often leading to prolonged periods of migrant work and reduced family contact. Economic hardships also adversely affect parents with relentless stress of making ends meet and pessimism about the future. These pressures and negative emotions can be easily transmitted to their children. Moreover, families struggling with finances often overlooked appropriate parenting styles. When the existing parenting styles clash with children's psychological and emotional needs, it breeds estrangement between children and parents, thereby further impairing their psychological and emotional wellbeing. This finding is consistent with Karl Marx's argument that the economic base shapes its superstructure in structural, cultural, and ideological forms (72).

Theme 3: Grade obsession is at the center of student wellbeing crisis.

Since 2021, China has implemented the “Double Reduction” policy, aiming to alleviate grade obsession and academic stress by restricting homework and extracurricular tutoring in primary and secondary schools. Despite rigorous enforcement of this policy, our findings indicate that the “Score-Is-All” mindset remains deeply ingrained in Chinese society. Students were indoctrinated with score-centric criteria for success or failure from an early age, while teachers and parents consistently instilled anxiety about failure through comments like, “You must attend high school and college,” “Your parents have sacrificed everything for you,” and “Hard work alone will guarantee success.” Consequently, the fear of failure overshadows any intrinsic motivation or personal interests that might otherwise drive students' holistic development. As one ninth-grade girl complained, “I am good at singing and dancing, but I guess that doesn't matter because I failed at math.”

Another significant consequence of grade obsession is the diminished self-esteem experienced by low-achieving students. We found students' academic performance significantly impacted their social decisions, with those who underperformed academically often feeling that they were disliked by teachers and parents. This perception can induce a sense of inferiority, subsequently leading to a gradual social withdrawal characterized by a reluctance to engage in group activities and assume leadership roles. For example, when asked about his responsibility in class, a sixth-grade boy remarked, “only good students get to be group leaders... I don't want to do it, and it is not appealing to me.” Furthermore, low-achieving students reported a heightened level of anxiety related to their appearance that further contributes to their feelings of self-doubt and inadequacy.

## 5 Discussion

The present study investigated PWB and SEC among Chinese children and adolescents following 3 years of experiencing the COVID-19 pandemic to identify salient patterns, essential determinants, and interrelationships between the two constructs. Overall, about 22.4% and 18.5% of the participants were classified as having low-level PWB and SEC, respectively. These results are consistent with data obtained during the pandemic, which showed the prevalence of mental health problems and social-emotional dysfunctions ranging from 17% to 25% (34, 54, 73–75). The fact that PWB and SEC problems exist in a similar magnitude after the pandemic indicates the long-lasting impact of COVID-19 on the PWB and development of the youth population, as many researchers have argued (20, 76, 77).

### 5.1 Demographic factors

A notable finding from the study is that a student's gender and grade level have a significant impact on their PWB, but not on their SEC. Research in psychiatry consistently reports a higher risk of mental disorders among female adolescents, which has been attributed to factors such as interpersonal stressors, gender inequality, low self-esteem, and negative body image (17, 20, 78). Additionally, the present study corroborates a recent study on Israeli youth (17), which highlighted the vulnerability of 14- and 15-year-olds to mental disorders. Puberty-related changes, complex social relations, and academic pressures may have contributed to the pronounced low-level PWB among 8th and 9th graders (17, 79). However, the study revealed that gender and grade level do not significantly predict students' SEC. This could be due to cultural expectations and gender roles in Chinese society, which may potentially reduce gender-based disparities in SEC development. Furthermore, during childhood and adolescence, SEC is primarily influenced by familial and educational factors rather than solely by demographic factors such as gender and age (80, 81).

However, a student's only child status appears to have limited influence on his/her PWB and SEC. The lack of difference in PWB and SEC between only children and those with siblings might be attributed to the strong extended family linkages emphasized in Chinese tradition (82, 83): Cousins from the extended families play a sibling-like role that bridges the gap left by the absence of siblings. By living nearby and growing up together, the presence of cousins can offset the social and emotional deficits facing singleton children but can also introduce mental health challenges such as peer pressure and sibling rivalry. This unique observation offers insights into how technology and social tradition can influence PWB and SEC for the younger generations.

### 5.2 Academic-related factors

Academic rank among students was identified as a key factor in predicting PWB and SEC, which aligns with previous findings (84–86). This might be rooted in the Chinese societal belief that academic excellence holds high value, with both parents and children believing that knowledge changes destiny (87, 88). This perception can cause students with lower academic standings to feel overwhelmed and uncertain about their future (89). Moreover, students who achieve good grades tend to possess stronger self-efficacy and emotional management skills, which enable them to adapt more easily to their environment, effectively cope with negative emotions, and develop positive peer relationships (90, 91), thus leading to high-level PWB and SEC.

The differences in PWB and SEC across school locations followed a non-linear pattern, with county schools showing the highest levels and township schools representing the most vulnerable context for students' PWB and SEC development. On the one hand, township schools fall behind county schools in educational resources and psychological support services (92). On the other hand, compared with village communities, townships display weaker social cohesion, with less close-knit extended families and neighborhood networks, which restricts students' access to external emotional support (93).

### 5.3 Socio-economic factors

Students' family life strongly influences their PWB and SEC. Children and adolescents from families with divorce or marital discord often face heightened psychological distress and lower SEC caused by long-term emotional stress (94, 95). Oldfield et al. (96) also noted that a fragile emotional bond with parents can lead to behavioral and emotional challenges, which can cause difficulties in social awareness, empathy, and self-reflection. While household economic status can influence children's wellbeing, we found its impact to be minor, possibly due to China's successful poverty alleviation initiatives in recent years, which ensure rural families have adequate means for basic needs (97–99).

Surprisingly, a student's left-behind status appears to have limited influence on his/her PWB and SEC. Rapid advances in communication technologies, such as video calls and social media, allow left-behind children to maintain regular contact with their parents, thereby reducing the negative emotions caused by separation (100, 101). Moreover, during the COVID-19 pandemic, extended home confinement allowed many parents to spend more time with their children, thereby reducing parent–child detachment and psychological distance (102). For some families, parental labor migration improved economic conditions and enhanced children's living standards and psychological security, which may have attenuated the overall negative effects of left-behind status.

### 5.4 Relationships between PWB and SEC

Our investigation into the relationship between the dimensions of PWB and SEC revealed that self-management shows the most prominent correlation with PWB, followed by self-awareness. This aligns with previous research indicating that self-management positively predicts and enhances mental health (103), which has been supported by several research studies (104–106). Self-management, involving the use of behavioral techniques for self-regulation (107), fosters improved learning autonomy and emotional regulation, which contribute to higher levels of PWB through enhanced agency, self-esteem, and stress management (108–110). The positive relationship between self-awareness and PWB is attributed to its role in promoting self-understanding, self-acceptance, and help-seeking behaviors. Self-awareness deepens individuals' understanding of their thoughts, emotions, and behaviors, which helps them to cope with challenging situations. Additionally, it nurtures self-acceptance, encouraging individuals to compassionately embrace their strengths and weaknesses, thereby mitigating the adverse effects of stress and adversity while bolstering resilience and emotional stability. Moreover, high self-awareness allows prompt identification of changes in mental health, facilitating timely intervention and appropriate assistance to prevent worsening mental health issues. Understanding the relationships of these SEC dimensions with PWB underscores the importance of nurturing these competencies for individuals' overall wellbeing.

Notably, the correlation between social awareness and PWB was the weakest among the variables examined. Students who perform well in social behavior may not necessarily attain good mental health levels. A potential explanation for this outcome is the development of a social acting persona among certain students. In such cases, there is a divergence between students' inner thoughts and their outward social conduct. These students often exhibit no overt signs of psychological distress; rather, they mask their emotions, which potentially leads to the development of more profound psychological issues that may remain concealed from the awareness of both parents and teachers.

### 5.5 Specific influences of post-pandemic context

Our research findings revealed several ongoing influences of the COVID-19 pandemic on PWB and SEC among Chinese children and adolescents. First, the most noticeable influence is the educational disruption during the pandemic and the efforts of schools to recover learning loss afterwards (e.g., longer study hours, heavier homework, frequent exams). The decrease in performance and the increase in workload experienced by the students during the post-pandemic era inevitably led to heightened performance anxiety and academic stress, which were found to detrimentally impact their psychological and emotional wellbeing (111, 112).

Second, the school closures and home quarantine measures implemented during the pandemic had increased the time that parents and children spent together, thereby exerting a deep influence on parent-child relationships. However, we found that the increased parental involvement did not necessarily translate into positive contributions to children's PWB, particularly when parental behavior is characterized by high control and rigorous demands. This observation aligns with the conclusions drawn by Zhao et al. (113). As argued by Wang and Eccles (114), conflicts between parenting styles and children's psychological and emotional needs can lead to detachment and resentment, subsequently hampering the wellbeing of children.

Third, economic downturns during the post-pandemic era exert considerable pressure on families, which can be readily transmitted to children, adversely influencing their PWB and SEC. As highlighted by Fegert et al. (115), prevalent financial risk factors that hamper children's wellbeing include parental unemployment, income reduction, and unmanageable household debt. Moreover, our research findings reveal that financial strains within families can lead to prolonged separations of family members due to heightened periods of migrant work, which can lead to a cascade of consequences for family relationships and structures. These findings align with existing literature on the COVID-19 pandemic, which indicates that its detrimental effects on students' academic achievements and PWB will persist for an extended duration (115, 116).

Lastly, the pandemic has introduced a positive change by accelerating the growth of social media and online communities where students can make friends, express opinions, and seek emotional support. Additionally, compared to the pre-pandemic period, parents also relaxed their control over their children's use of electronic devices. Those changes have provided Chinese youth with increased social opportunities and diverse experiences in the online space, thereby mitigating, to some degree, the adverse effects of the pandemic on their social and emotional outcomes. This finding is inconsistent with previous research that emphasized the negative impact of the pandemic on SEC development (117, 118).

## 6 Conclusions

This study examined the post COVID-19 status of PWB and SEC in China. Overall, it has been observed that students' PWB and SEC levels manifest at a moderate level, with discernible disparities noted among various baseline characteristics. Notably, significant differences in both PWB and SEC were observed based on factors such as students' grade level, academic rank, school location, parent marital status, household economic condition, and left-behind status. This investigation has also identified essential determinants influencing PWB and SEC, with specific emphasis on academic rank, parent marital status, and household economic condition. The interrelationships between different dimensions of PWB and SEC underscore the importance of self-management and self-awareness in enhancing the wellbeing of Chinese youth.

### 6.1 Practical implications

Based on the research findings, several implications can be proposed. Teachers in primary and secondary educational settings must remain vigilant in monitoring both the academic performance and emotional development of their students throughout their daily routines, especially for students with poor academic achievement, a disadvantaged socioeconomic background, and unstable parental relationships. In addition, we posit that non-left-behind children also warrant attention, particularly those with high social awareness. Parental relationships exert a substantial influence on students' mental health, so parents should also dedicate attention to their children's psychological issues, engage in timely communication with the school, and refrain from adhering to an “academic supremacy” mindset. Researchers should collaborate with schools and employ technological tools to devise appropriate teaching interventions to enhance students' self-management and self-awareness, thereby improving their mental health.

### 6.2 Limitation and future research agenda

Several limitations of our research study should be noted before interpreting the research results. First, this study was conducted in a city in China, and the research results may not be representative of other provinces and cities. Second, the source of the questionnaire data was self-reported based on students' own perception of their school life, which runs the risk of response bias. Questions may therefore remain about data validity. The questionnaire may also have been somewhat challenging for young children to comprehend. Future research should therefore seek more appropriate methods for assessing SEC, such as group collaborative activities, and use qualitative data to aid in the interpretation of study results. Third, the duration of this study was relatively short, and the data obtained can only represent the students' PWB and SEC levels in the short term. To gain a more comprehensive understanding, future research should use longitudinal designs to track students' long-term PWB and SEC patterns. Additionally, technological platforms could be employed to interventions with students with mental health issues and investigate the effectiveness of such interventions (41).

## Data Availability

The datasets presented in this study can be found in online repositories. The names of the repository/repositories and accession number(s) can be found in the article/Supplementary material.
