# Supplementary material for: Psychological wellbeing and social emotional competence of Chinese children and adolescents in the post-pandemic era: patterns, determinants, and interrelations
Source: Front Public Health. 2025 Nov 18;13:1677632. doi: 10.3389/fpubh.2025.1677632 (PMC12668964; doi:10.3389/fpubh.2025.1677632)
Supplement: Supplementary file 1 [file Data_Sheet_1.pdf]

Supplementary Material1

Survey questionnaire

Greetings! Thank you for agreeing to participate in this survey. We are a research team from the Collaborative-Innovation Center of Informatization and Balanced Development of Basic Education, Central China Normal University. The survey questionnaire aims to obtain your basic information and your perception of your recent school life. This questionnaire is not an exam and will not be graded nor will it be accessed by your teachers, parents, or classmates. Filling out the questionnaire is entirely voluntary. Please answer truthfully based on your own situation. Thank you!

- **Part 1: Basic information (13 items)**

1. Name____________
2. Age_____________
3. Location______________
4. School name____________
5. Name of class teacher_____________
6. Gender: a. Male b. Female
7. Grade:

   a. 3 b. 4 c. 5 d. 6 e. 7 f. 8 g. 9

8. Please fill in the number of your brothers and sisters
Elder brother(s)______, elder sister(s)______, younger brother(s)______, younger sister(s)______
9. What is the relationship between your father and mother?
a. Married b. Divorced c. Other
10. Which of the following situations best describes your family's current situation?
a. My father works outside the house, and my mother takes care of the household.
b. My mother works outside the house, and my father takes care of the household.
c. Both parents work outside.
d. Both parents are at home.
e. Other____________
11. Which of the following descriptions best describes your living condition?
(1) I have a private room.
a. Yes b. No
(2) I have a desk for studying.
a. Yes b. No
(3) I have a computer which I can use to study.
a. Yes b. No
12. Please evaluate your grades in math
a. Excellent b. Good c. So-so d. Poor
13. Please evaluate your grades in Chinese
a. Excellent b. Good c. So-so d. Poor

- **Part 2: Social emotional competence scale (25 items)**

Self-awareness

1. I know what I am thinking and doing.
2. I understand what I do and why I do it.
3. I understand my moods and feelings.
4. I know when I am moody.
5. I can read people’s faces when they are angry.

Social Awareness

6. I can recognize how people feel by looking at their facial expressions.
7. It is easy for me to understand why people feel the way they do.
8. If someone is sad, angry, or happy, I believe I know what they are thinking.
9. I understand why people react the way they do.
10. If a friend is upset, I have a pretty good idea why.

Self-management

11. I can stay calm in stressful situations.
12. I can stay calm and overcome anxiety in new or changing situations.
13. I can stay calm when things go wrong.
14. I can control the way I feel when something bad happens.
15. When I am upset with someone, I will wait till I have calmed down before discussing the issue.

Relationship Management

16. I will always apologize when I hurt my friend unintentionally.
17. I always try and comfort my friends when they are sad.
18. I try not to criticize my friend when we quarrel.
19. I am tolerant of my friend’s mistakes.
20. I stand up for myself without putting others down.

Responsible Decision-Making

21. When making decisions, I take into account the consequences of my actions.
22. I ensure that there are more positive outcomes when making a choice.
23. I weigh the strengths of the situation before deciding on my action.
24. I consider the criteria chosen before making a recommendation.
25. I consider the strengths and weaknesses of the strategy before deciding to use it.

- **Part 3: Psychological well-being scale (25 items)**

Positive effect

1. I am proud because someone complimented me on something I had done.
2. I am particularly excited or interested in something.
3. I am pleased about having accomplished something.
4. Things have been going my way.

Agitation scale

5. I often get mad and say nasty things.
6. I sometimes get so mad that I kick things or break things.
7. Lately, I’ve been kind of grouchy.
8. I sometimes intentionally threaten my friends.
9. I sometimes feel like a powder keg ready to explode.

Depression

10. I feel so blue or depressed that it interfered with my daily activities.
11. I often suffer from nervousness, which appears as irritability, irritability, or anxiety.
12. It seems as if nothing turned out the way I wanted it to.
13. I feel somewhat apart or alone, even with friends.
14. Personal worries have been getting me down physically—that is, making me ill.

Meaninglessness

15. I feel all alone in the world.
16. My life seems empty.
17. I do not know what to do with my life.
18. I feel as if my life just is not complete.
19. I feel as if I am not interested in anything.

Self-esteem

20. I feel that I am a person of worth, at least equal to others.
21. At times I think I am no good at all.
22. I feel I do not have much to be proud of.
23. On the whole, I am satisfied with myself.
24. All in all, I tend to feel that I am a failure.
25. I feel worthless at times.
