# Supplementary material for: Psychological wellbeing and social emotional competence of Chinese children and adolescents in the post-pandemic era: patterns, determinants, and interrelations
Source: Front Public Health. 2025 Nov 18;13:1677632. doi: 10.3389/fpubh.2025.1677632 (PMC12668964; doi:10.3389/fpubh.2025.1677632)
Supplement: Supplementary file 2 [file Data_Sheet_2.pdf]

Supplementary Material2

Complete results of multiple logistic regression analysis

Table 1 Regression results for predicting psychological well-being (PWB)

	B	SE	Wald	*p*	Exp(B)	95% CI	
						lower	upper
**Low PWB**							
Intercept	-1.262	.520	5.882	.015			
Sex							
Male	-.420	.112	14.141	**<.001**	.657	.528	.818
Female	0^b						
Grade							
3-4	-.683	.185	13.613	**<.001**	.505	.351	.726
5-6	-.597	.176	11.550	**.001**	.550	.390	.777
7	-.784	.151	27.073	**<.001**	.456	.340	.613
8-9	0^b						
School location							
Village	-.044	.240	.033	.856	.957	.598	1.533
Township	.359	.154	5.434	**.020**	1.432	1.059	1.937
County	0^b						
Academic rank							
Poor	2.604	.327	63.579	**<.001**	13.516	7.127	25.634
Below average	1.918	.257	55.873	**<.001**	6.808	4.117	11.258
Above average	.964	.205	22.118	**<.001**	2.621	1.754	3.916
Excellent	0^b						
Left-behind status							
Father employed	-.120	.462	.068	.794	.887	.359	2.192
Mother employed	-.562	.516	1.185	.276	.570	.207	1.568
Double left behind	-.128	.457	.078	.780	.880	.359	2.154
Non-left behind	-.390	.462	.710	.399	.677	.274	1.676
Other	0^b						
Parent marital status							
Other	1.183	.257	21.172	**<.001**	3.264	1.972	5.402
Divorced	.613	.182	11.290	**.001**	1.846	1.291	2.640
Married	0^b						
Household economic condition							
Low	1.096	.265	17.168	**<.001**	2.993	1.782	5.027
Medium	.433	.126	11.835	**.001**	1.542	1.205	1.975
High	0^b						
Only child status							
Yes	-.089	.116	.596	.440	.914	.729	1.148
No	0^b						
**Middle PWB**							
Intercept	-.358	.441	.658	.417			
Sex							
Male	.001	.090	.000	.988			
Female	0^b						
Grade							
3-4	-.099	.147	.454	.500	.906	.679	1.208
5-6	-.325	.145	4.977	**.026**	.723	.544	.961
7	-.388	**.127**	9.396	**.002**	.678	.529	.869
8-9	0^b						

	B	SE	Wald	*p*	Exp(B)	95% CI	
						lower	upper
School location							
Village	.398	.179	4.941	**.026**	1.489	1.048	2.116
Township	.366	.133	7.606	**.006**	1.443	1.112	1.872
County	0[b]						
Academic rank							
Poor	1.578	.287	30.149	**<.001**	4.844	2.758	8.508
Below average	1.267	.201	39.559	**<.001**	3.550	2.392	5.268
Above average	.670	.136	24.156	**<.001**	1.954	1.496	2.553
Excellent	0[b]						
Left-behind status							
Father employed	-.120	.462	.068	.794	.887	.359	2.192
Mother employed	-.562	.516	1.185	.276	.570	.207	1.568
Double left behind	-.128	.457	.078	.780	.880	.359	2.154
Non-left behind	-.390	.462	.710	.399	.677	.274	1.676
Other	0[b]						
Parent marital status							
Other	1.183	.257	21.172	**<.001**	3.264	1.972	5.402
Divorced	.613	.182	11.290	**.001**	1.846	1.291	2.640
Married	0[b]						
Household economic condition							
Low	1.096	.265	17.168	**<.001**	2.993	1.782	5.027
Medium	.433	.126	11.835	**.001**	1.542	1.205	1.975
High	0[b]						
Only child status							
Yes	-.089	.116	.596	.440	.914	.729	1.148
No	0[b]						

Note: SE, standard error; CI, confidence interval; Double-left-behind refers to children and adolescents whose parents are both migrant workers away from home, and b refers to the baseline standard used for comparison with other variables.

Table 2 Regression results for predicting social emotional competence (SEC)

	B	SE	Wald	*p*	Exp(B)	95% CI	
						lower	upper
**Low SEC**							
Intercept	.853	.413	4.277	.039			
Sex							
Male	.128	.089	2.072	.150	1.137	.955	1.354
Female	0[b]						
Grade							
3-4	.172	.144	1.415	.234	1.187	.895	1.576
5-6	.080	.142	.315	.575	1.083	.820	1.430
7	.106	.125	.717	.397	1.112	.870	1.421
8-9	0[b]						
School location							
Village	-.119	.184	.416	.519	.888	.619	1.274
Township	-.303	.132	5.275	**.022**	.739	.570	.957
County	0[b]						
Academic rank							
Poor	-1.500	.310	23.415	**<.001**	.223	.122	.410

	B	SE	Wald	p	Exp(B)	95% CI	
						lower	upper
Below average	-1.226	.195	39.362	**<.001**	.293	.200	.430
Above average	-.677	.128	27.788	**<.001**	.508	.395	.653
Excellent	0^b						
Left-behind status							
Father employed	-.134	.378	.125	.724	.875	.417	1.834
Mother employed	.155	.420	.136	.713	1.167	.513	2.656
Double left behind	-.345	.374	.850	.356	.708	.340	1.475
Non-left behind	-.218	.377	.335	.563	.804	.384	1.684
Other	0^b						
Parent marital status							
Other	-.264	.231	1.306	.253	.768	.488	1.208
Divorced	-.335	.164	4.173	**.041**	.715	.519	.986
Married	0^b						
Household economic condition							
Low	-.776	.233	11.054	**.001**	.460	.291	.727
Medium	-.525	.097	29.270	**<.001**	.592	.489	.716
High	0^b						
Only child status							
Yes	-.006	.093	.004	.948	.994	.828	1.194
No	0^b						
**Middle SEC**							
Intercept	-2.356	.502	22.014	0.000			
Sex							
Male	.063	.108	.334	.563	1.065	.861	1.317
Female	0^b						
Grade							
3-4	.090	.187	.235	.628	1.095	.759	1.578
5-6	.104	.182	.328	.567	1.110	.777	1.585
7	-.103	.148	.487	.485	.902	.675	1.205
8-9	0^b						
School location							
Village	.034	.210	.027	.870	1.035	.685	1.563
Township	.308	.151	4.155	**.042**	1.360	1.012	1.828
County	0^b						
Academic rank							
Poor	1.992	.314	40.233	**<.001**	7.328	3.960	13.560
Below average	1.070	.291	13.528	**<.001**	2.915	1.648	5.156
Above average	.784	.262	8.944	**.003**	2.189	1.310	3.659
Excellent	0^b						
Left-behind status							
Father employed	.056	.401	.019	.890	1.057	.482	2.322
Mother employed	.000	.460	.000	.999	1.000	.406	2.462
Double left behind	.029	.393	.005	.942	1.029	.477	2.221
Non-left behind	-.095	.401	.056	.813	.909	.414	1.997
Other	0^b						
Parent marital status							
Other	.407	.221	3.378	.066	1.502	.973	2.317
Divorced	.151	.170	.791	.374	1.163	.833	1.624
Married	0^b						
Household economic condition							
Low	.875	.232	14.248	**<.001**	2.398	1.523	3.776
Medium	.433	.141	9.401	**.002**	1.541	1.169	2.032
High	0^b						

	B	SE	Wald	*p*	Exp(B)	95% CI	
						lower	upper
Only child status							
Yes	-.139	.116	1.432	.231	.871	.694	1.092
No	0^b						

Note: SE, standard error; CI, confidence interval; Double-left-behind refers to children and adolescents whose parents are both migrant workers away from home, and b refers to the baseline standard used for comparison with other variables.
