# Supplementary material for: Psychological wellbeing and social emotional competence of Chinese children and adolescents in the post-pandemic era: patterns, determinants, and interrelations
Source: Front Public Health. 2025 Nov 18;13:1677632. doi: 10.3389/fpubh.2025.1677632 (PMC12668964; doi:10.3389/fpubh.2025.1677632)
Supplement: Supplementary file 4 [file Data_Sheet_4.zip › Supplementary Material 4/Student 1-1A_Anonymized.docx]

# Interview Notes: Student 1-1A

Student1-1 Home Visit Report

Date and Time: July 1, 2023, 10:00–11:00 a.m.
Participants: the interviewer, the interviewer, and Chinese teacher the interviewer

The family member we contacted for Student1-1 was her grandmother. Even over the phone, she was very warm and enthusiastic about our visit, insisting on inviting us for lunch and preparing many fruits and drinks. The grandmother looked quite young, around 55 years old, and was dressed in a black dress. She seemed rather familiar with the interviewer the interviewer, suggesting they often communicate. On the way there, the interviewer the interviewer kept praising Student1-1, saying that she was a sensible and intelligent child who always watched the teacher attentively in class, with bright, focused eyes.

We drove to a levee on the outskirts of the city, where there was a row of small house-like storefronts. Student1-1’s family rented one of the ground-floor units. Her grandmother greeted us on the levee, led us down the stone steps through the courtyard, and into the house. Student1-1 greeted us warmly and confidently. The home was small but neat and tidy, and she had her own bedroom (which used to belong to her parents), so the living environment was quite acceptable.

*Through our conversation with the grandmother, we learned that Student1-1’s mother has been working in a metropolitan city in eastern the country. About a year ago, her parents had a big argument, after which her father left home in anger to work in another city. They are now separated. Whether they are divorced remains unclear—although Student1-1 wrote “divorced” in the questionnaire, her grandmother believes it was just a temporary quarrel and still hopes they will reconcile one day. Overall, both parents visit rarely, especially the father, who has almost disappeared from her life (according to the interviewer the interviewer, he married into the mother’s family).*

*When talking about her father, Student1-1 seemed casual and indifferent but mentioned softly, “He used to say he missed me…” She then refused to elaborate further, saying she had deleted their chat records. Her mother is more involved—during summer vacations, she often takes Student1-1 to a metropolitan city in eastern the country. In two weeks, she will go again, and it was clear she was looking forward to it. However, during the conversation, she also expressed some resentment about her parents’ long absence. She said the clothes they bought her never fit: “They don’t even know how tall I am now… maybe not even which grade I’m in.”*

When discussing her low emotional scores in the questionnaire, Student1-1 explained that she had indeed been in a bad mood at that time because of her parents’ separation, and her grades had declined, which made her feel depressed. But now she has adjusted, thanks to a close friend she can confide in, who helped her through the tough period. Her grades have also improved (she said she subscribed to a learning app on her own and learned independently with good results).

My strongest impression of Student1-1 is that she is more mature and self-motivated than her peers. For example, she asked her family to recharge money for the app membership because she felt it was helpful. In second grade, she saw a cat she liked and decided to buy it on her own. [Perhaps because her parents are away, she has learned to make her own decisions early. Since she performs well in school and expresses herself clearly, her grandmother tends to give her freedom. This might be a good thing—overly strict or controlling parenting often causes more problems.]

*Student1-1 is quite outgoing, unafraid of talking to strangers, and willing to share her thoughts with us. According to her grandmother, she even sings karaoke along the levee during walks—quite bold for a ten-year-old. When asked about her dream career, she said she wanted to host a TV show alongside “He Jiong”, so perhaps she is already practicing her communication skills and enjoying the process.*

[Overall, I believe Student1-1’s risk level is not very high. The emotional instability shown in her questionnaire might have been temporary. Although her family background—left-behind child with separated parents—fits the profile of a high-risk group, she demonstrates strong psychological resilience and seems to be coping well.]
