# Supplementary material for: Psychological wellbeing and social emotional competence of Chinese children and adolescents in the post-pandemic era: patterns, determinants, and interrelations
Source: Front Public Health. 2025 Nov 18;13:1677632. doi: 10.3389/fpubh.2025.1677632 (PMC12668964; doi:10.3389/fpubh.2025.1677632)
Supplement: Supplementary file 4 [file Data_Sheet_4.zip › Supplementary Material 4/Student 1-1B_Anonymized.docx]

# Interview Notes: Student 1-1B

Student1-1 Home Visit Report

Date and Time: July 1, 2023, 10:00–11:00 a.m.
Participants: the interviewer, the interviewer, and Chinese teacher the interviewer

The person we contacted for Student1-1’s home visit was her grandmother. Even over the phone, she sounded warm and enthusiastic, repeatedly insisting on inviting us for lunch and preparing plenty of fruits and drinks. The grandmother looked relatively young, around 55 years old, wearing a black dress, and seemed familiar with the interviewer the interviewer, suggesting that they often communicate. On the drive over, the interviewer the interviewer spoke highly of Student1-1, praising her as a sensible and intelligent child who listens attentively in class, with eyes full of focus and curiosity.

We drove to a levee on the outskirts of the city, where a row of small, shop-like houses stood. Student1-1’s family rented one of the ground-floor units. Her grandmother greeted us on the levee and led us down the stone steps through a courtyard to the entrance. Student1-1 greeted us politely and confidently. The home was small but tidy and well-kept. She even had her own bedroom (formerly her parents’ room), so the living conditions were decent.

*Through casual conversation, we learned that Student1-1’s mother has been working in a metropolitan city in eastern the country. About a year ago, her parents had a major argument, after which her father left in anger to work in another city. They are now separated. Whether they are divorced is unclear; although Student1-1 indicated “divorced” on her questionnaire, her grandmother believes it was just a temporary conflict and hopes they will reconcile one day. In any case, both parents seldom visit, especially the father, who has almost disappeared from her life (according to the interviewer the interviewer, he married into the mother’s family).*

*When talking about her father, Student1-1 appeared indifferent but briefly said, “He used to say he missed me…” before dropping the topic, noting that she had deleted those chat records. Her mother is more involved in her life and often brings her to a metropolitan city in eastern the country during summer breaks. Student1-1 said she helps her mother with work there and looked forward to their upcoming trip, excitedly mentioning plans to visit a cat café and a water park. She clearly enjoys spending time with her mother and appreciates their harmonious relationship.*

*However, she also revealed some frustration about her parents’ long absence. She mentioned, “The clothes they buy for me never fit—they don’t even know how tall I am now… maybe not even which grade I’m in.” Regarding her low emotional scores in the questionnaire, Student1-1 explained that she had been feeling upset at the time due to her parents’ separation and declining grades. Now, she feels better thanks to a close friend who supported her through that difficult period. Her grades have improved too, partly because she purchased a learning app membership on her own initiative and found it helpful.*

She also shared her coping mechanism for sadness—eating a lot of sweets, cakes, and ice cream until she sometimes got stomachaches. Though not a healthy outlet, it reflects a young child’s limited ways to express and release emotions. Fortunately, her friend’s companionship later helped her recover.

*During our conversation, it was evident that Student1-1 is a highly self-motivated and humble child. When she once ranked first in her grade, she said she didn’t feel overly excited and simply asked her grandmother to buy her a cake as a small reward. She remarked, “If ten people get full marks, then getting full marks just makes you the 11th. You shouldn’t get proud just because someone praises you—you should stay humble.” Hearing such words from a ten-year-old was both surprising and touching—surprising for her maturity, and comforting to see how well she has grown under her grandmother’s care.*

When asked about hobbies, Student1-1 said she plays video games but only after finishing her homework, showing strong self-discipline likely developed from early independence. Most of her gaming companions are boys, as she prefers the types of games they play. She also mentioned having some online friends but emphasized that she only chats with girls and never with unfamiliar boys—showing an impressive awareness of online safety for her age.

*Still, her interest in “boys’ games” sometimes makes her feel isolated: she can’t play basketball with boys or jump rope with girls, which makes her feel awkward at times. Yet, she remains cheerful, well-liked, and capable of self-regulation and self-acceptance.*

*Interestingly, she said she prefers going to school over staying home during vacations because she enjoys the structured, purposeful routine. She had already completed half of her summer homework just a week into the break. On school days, she usually gets home around 5:30 p.m. and goes to bed by 9:00, though she often can’t fall asleep right away—sometimes staying awake until 10 or 11 p.m., and once even until 4 a.m. She didn’t specify what she was doing then—perhaps gaming or watching a “metaverse-style” livestream she mentioned, which she discusses with classmates.*

*Another detail raised some uncertainty: Student1-1 said she remembers little about her father, only his face and “big belly.” Though she said this casually, it hinted at past hurt. She added that she chats with her “uncle.” Initially, we thought she meant her mother’s brother, but on reflection, it might be her mother’s current partner in a metropolitan city in eastern the country. She described him as someone “who plays Egg Party (a game) and helps Mom with chores.” This suggests that Student1-1 understands more than adults may assume.*

Overall, Student1-1 left a deep impression of maturity and self-direction. She makes her own decisions—like buying a cat in second grade or subscribing to an app she believed would help her study—and her grandmother generally respects her choices. This autonomy seems beneficial, given her competence and strong sense of responsibility. She’s also sociable and expressive, unafraid to talk with strangers and eager to share her thoughts. Her grandmother even mentioned that she likes to sing karaoke outdoors along the levee—quite bold for a ten-year-old!

*When asked about her dream job, she said she wants to be a TV host like “He Jiong”, perhaps already preparing herself by practicing confidence and communication.*

*In conclusion, although Student1-1’s family background—separation and partial parental absence—fits the profile of a “high-risk” child, she demonstrates remarkable emotional resilience, self-regulation, and independence. Her questionnaire responses likely reflected a temporary downturn, not a long-term issue. She is a bright, capable, and emotionally intelligent child coping impressively well with her circumstances.*
