# Supplementary material for: Psychological wellbeing and social emotional competence of Chinese children and adolescents in the post-pandemic era: patterns, determinants, and interrelations
Source: Front Public Health. 2025 Nov 18;13:1677632. doi: 10.3389/fpubh.2025.1677632 (PMC12668964; doi:10.3389/fpubh.2025.1677632)
Supplement: Supplementary file 4 [file Data_Sheet_4.zip › Supplementary Material 4/Student 1-2C_Anonymized.docx]

# Interview Notes: Student 1-2C

Student Name: Student1-2
Grade: Fourth Grade
Interviewers: Director Guo, C, D, and the accompanying homeroom teacher

We drove into a narrow alley surrounded by residential buildings. After climbing eight flights of stairs, we arrived at Student1-2’s aunt’s home. The aunt had specially called the father, who works in a nearby town, to come home and join the conversation.

1. Family Background

The parents are divorced, and the child has been raised by the aunt since early childhood. The aunt has her own children but cares deeply for Student1-2. The father works in a township and is usually very busy, while the mother seldom communicates with the child.

2. Relationships and Social Interaction

According to the homeroom teacher, Student1-2 has a few good friends at school and generally gets along well with classmates.

3. Academic Performance and Behavior

The student’s grades are average. He is obedient and responsible—he even knows how to cook. The aunt mentioned that if she hasn’t prepared a meal, he will take the initiative to make dumplings for her. Student1-2 said that he learned to cook because he wants to be able to take care of himself in the future and become more capable.

4. Learning Support

*The father said he cannot provide much academic guidance himself, so he prefers to pay for tutoring: “If it’s helpful for his studies, we’ll do it. He’s been going to tutoring for a while, but his progress is still quite slow.” His math grades are relatively better, usually scoring above 80.*

The father also mentioned that the child has poor study habits—he doesn’t take the initiative to do homework and likes to play on his phone. He attends tutoring classes from Monday to Friday and plays on his phone during weekends. (This differs from the child’s own description—he said that no one restricts his phone use, and he plays for about five to six hours a day.)

5. Personality and Long-term Development

*When in a bad mood, Student1-2 tends to be irritable. According to his father, when he loses in a game, he gets upset and may cry in frustration. The father tries to comfort him but adds, “If he keeps getting angry, I’ll stop him from playing games. He’s been like this since he was little.”*

Regarding future expectations, the father hopes that his child will stay both physically and mentally healthy and perform well in school. He doesn’t have any particular hobbies, though he plays table tennis quite often.

The aunt mentioned that there have been some conflicts between Student1-2 and his female desk-mate. The child once asked the aunt to contact the classmate’s parents and teachers, but the aunt felt it was a small issue and that teachers were busy, so she encouraged him to handle it himself.

6. Additional Observations

*The aunt mentioned that Student1-2 once wrote a very good essay titled “A Letter to My Mother.” In it, he expressed resentment toward his mother for being away and not caring for him. However, even with his father—who tries to chat with him daily—the conversations usually end quickly, as the child rarely opens up or takes initiative to communicate. He seems sensitive but not good at expressing emotions.*

7. Interviewer’s Reflections

*Overall, the main issues observed with Student1-2 are emotional instability and difficulty managing anger. He rarely communicates with family members, tends to close himself off, and spends excessive time immersed in the virtual world. His long-term separation from his mother appears to be a major contributing factor. The father, who often works away from home, believes that providing financial support is sufficient and thus spends little time with the child. As a result, the aunt has taken on the “mother” role—but emotional substitution cannot replace the affection he truly longs for.*

*Additionally, I noticed a sign on Student1-2’s bedroom door that read “No entry for children under eight.” Upon inquiry, I learned that when he was younger, he used to follow his older brother around, and the brother had written the sign to keep him out. The child said, “I didn’t know what I was feeling then; I just wanted to play with him.” It’s uncertain whether this incident has left any emotional impact on him.*

Student1-2 also mentioned that he plays Honor of Kings (a mobile game) and admitted that in extreme situations, he sometimes types angry messages to scold teammates.

Summary:

Student1-2 demonstrates signs of emotional volatility, social withdrawal, and heavy reliance on digital entertainment. While he receives some care from his aunt and financial support from his father, the lack of consistent parental presence and emotional connection may be influencing his temperament and self-regulation.
