# Supplementary material for: Psychological wellbeing and social emotional competence of Chinese children and adolescents in the post-pandemic era: patterns, determinants, and interrelations
Source: Front Public Health. 2025 Nov 18;13:1677632. doi: 10.3389/fpubh.2025.1677632 (PMC12668964; doi:10.3389/fpubh.2025.1677632)
Supplement: Supplementary file 4 [file Data_Sheet_4.zip › Supplementary Material 4/Student 2-4_Anonymized.docx]

# Interview Notes: Student 2-4

Student 2-4

the interviewers:the teacher Guo, the interviewer B, the teacher Song

When we arrived at Student 2-4's house, both her parents were home, but Student 2-4 wasn't. Her mother said she'd been called and would be right back. Since the class teacher was there, she and the parents discussed the child's situation. In their eyes, Student 2-4 loved playing with her phone, even at workshops and sports meets. The homeroom teacher, realizing she enjoyed helping her, assigned her to help collect group homework. However, a mishap occurred the next day, when she failed to collect several books, and was subsequently told not to do the job. Her parents suspected Student 2-4 wasn't very happy at her grandmother's house (her grandmother and grandfather were a reconstituted family, so her grandmother wasn't her biological grandmother), feeling that being at her grandmother's house was different from being at her own. At this point, we, including the homeroom teacher, learned that while her mother picked her up from school every day, it was her grandmother who did the homework and checking, as she was a teacher at a local middle school.

*After spending some time with her parents at home, Student 2-4 returned, accompanied by her biological grandmother and her granddaughter from her second husband, a slightly older woman in the same grade. We went to her study to chat privately. There was a computer on the desk, and books on the bookshelf, including "Camel Xiangzi" and "The Little Prince." But after asking, I learned that although this was her study, she didn't do her homework there, and the computer had long since broken down. Every day after breakfast at home, her mother would take her to school. Then, in the evening, she'd be picked up and sent to her grandmother's house to do her homework, where she'd check her work for every class. When asked about anything that made her happy recently, she mentioned that her grandmother wasn't home lately, so she could watch TV all the time. When asked about any stress, annoyance, or unhappiness, she said her grandmother was too strict and annoying. She felt her grandmother was too strict with her, and even if she made a slight improvement on a test, she'd still feel unhappy if she'd just say, "Well, I did well this time." Furthermore, she felt her grandmother was "too exaggerated" when criticizing her, "not just me, but also my parents for not managing me well enough." As she spoke, Student 2-4 pouted, tears glistening in her eyes. Her father is a truck driver, and recently injured his hand, so he's staying home. Her mother works as a loan and rent collector, and after picking her up from school, she cleans, washes clothes, and does chores. So why send her to her grandmother's house to study instead of her mother? I asked, because she's maintained this schedule since first grade. She said her grandmother is a teacher at a local middle school, and she thinks she can help better.*

*When she encounters something upsetting, or when her grandmother puts too much pressure on her, or criticizes her, she doesn't confront her directly, doesn't confide in her friends, and rarely tells her parents. "Usually," she said, raising her hand, palm up, then lowering it, palm down, in a "lucky" gesture, "I deal with it myself." Why not confide in her friends? I asked her curiously, and she said, "Friends have their own friends, and friends tell friends, and then one person tells another, and then ten, ten, a hundred, and eventually everyone knows." She said she'd experienced this before: she'd told her secret to a friend, who then passed it on to another friend, and finally to herself. She was furious, so she never shared such stories again. Furthermore, because her grandmother wouldn't allow her to go out with her classmates on weekends, she felt she didn't have any particularly close friends. "Because I never went out with them, our relationships gradually faded."*

*When asked about her hobbies besides studying, she said she loved skipping rope, and that her grandmother would ask her to do it every night after dinner. When asked further if she truly enjoyed skipping or if her grandmother had forced her to do it, her eyes flickered, her lips curled, and she shook her head, saying, "It was her grandmother who asked her to do it. She said it would be on the college entrance exam, so she told me to skip rope."*

*When asked what career she wanted to pursue in the future, she thought for a moment and said she wanted to be a police officer because she'd seen a lot of drug traffickers in the "Student World" magazine, and she wanted to catch them and eliminate this harm for the people. When asked about her perceived strengths, she shook her head. When asked about her shortcomings, she admitted she was a bit impatient, getting anxious when faced with something she couldn't do properly, whereas her older sister (mentioned above) wasn't like that.*

Overall, Student 2-4's grandmother's strict management of her put her under considerable mental pressure. She often related her grandmother to her happy and unhappy experiences. Furthermore, because she had experienced hurt feelings in her interactions with classmates and friends, she was reluctant to confide in or share even unhappy experiences, leading to a profound lack of interpersonal security and trust. She also rarely communicated with her parents, who didn't provide effective support in dealing with her grandmother's management. Therefore, she didn't feel a sense of security from them.

*After the interview, upon leaving the study, Student 2-4's father said, "We think you should just make mistakes now. We're your support." This suggests that her father is quite open-minded and supportive, but there was a lack of communication between the two sides. Later, while going downstairs, Student 2-4's parents were briefly advised to communicate more and address their child's sensitive emotions.*
