# Supplementary material for: Psychological wellbeing and social emotional competence of Chinese children and adolescents in the post-pandemic era: patterns, determinants, and interrelations
Source: Front Public Health. 2025 Nov 18;13:1677632. doi: 10.3389/fpubh.2025.1677632 (PMC12668964; doi:10.3389/fpubh.2025.1677632)
Supplement: Supplementary file 4 [file Data_Sheet_4.zip › Supplementary Material 4/Student 3-1_Anonymized.docx]

# Interview Notes: Student 3-1

Interview with Student 3-1’s Mother
the interviewers: the teacher 1, the interviewer, and the teacher 2 (the the teacher)

*At eight o’clock in the morning, after breakfast and gathering, we split into three groups. Together with the teacher 1 and the teacher 2, I walked to the a local community. From a distance, we saw a woman in a denim dress waving at us — she was Student 3-1’s mother. the interviewere had contacted her the night before and again that morning before setting out, so she came down to greet us. As we walked and talked, she told us that Student 3-1 was attending extracurricular classes that morning — a reading class and an English class until 12:30, followed by a dance class at 3:30 in the afternoon. She also mentioned that when her daughter heard the teachers were coming for a home visit, she asked, “Is it because I didn’t fill out the questionnaire properly?” and admitted, “I just filled it out randomly.”*

Entering the home, we saw a red velvet sofa set. On it sat a little girl with big eyes and two braids — this was Student 3-1’s younger sister, eight years old and in second grade. Next to the sofa was a wooden table topped with a thick piece of glass slightly larger than the table surface, serving as both tabletop and protector. the interviewerhen we talked about tutoring, the mother explained that Student 3-1 tends to procrastinate when studying. Her math grades are relatively good, but in Chinese exams she often cannot finish writing her essays. Although she can clearly express her ideas orally, her written expression is weak, so her mother enrolled her in a reading class to help improve it.

*Regarding social relationships, when asked whether Student 3-1 had close friends, her mother said that she often takes her younger sister along to classmates’ birthday parties. This suggests that she gets along well with her peers, though this is only an inference since we did not meet her in person. From her mother’s and sister’s comments, it is also clear that the two sisters share a close relationship — “Although they sometimes argue, after a while she’ll come to her sister and make peace.”*

I noticed a large box of colored pens (about 48 colors) on the sofa and a smaller box of 12 colors on the opposite table. Upon asking, we learned that both girls enjoy drawing and sometimes draw on the walls at home. Behind the sofa, the white wall was covered in black and red crayon lines — the mother said the younger sister drew those, while Student 3-1 had drawn a fish on the wall in her own bedroom. the interviewerith her consent, we entered the room and saw a simple red sketch of a fish drawn on the wall behind the door.

One wall in the living room had a pink Hello Kitty height chart. the interviewerhile we were talking, the younger sister ran over to measure herself, and the mother said that Student 3-1 was now even taller than she was. The mother is about 1.6 meters tall.

*the interviewerhen asked about what Student 3-1 usually does in her free time, the mother said she likes to watch short videos on her phone or tablet, especially cooking and food-related ones. “For example, videos about making beautiful cakes... Once she even made watermelon juice herself.” This suggests that she has good hands-on ability. the interviewerhen asked how she felt about her daughter making juice, the mother said, “I don’t mind what she does, as long as she cleans up afterward.”*

*Later, when we asked about her expectations for her daughter beyond academic performance, she said she hopes the child can take more responsibility for household chores, such as washing dishes. “Sometimes when I ask her to wash the dishes, she says, ‘Give me ten minutes,’ and then she really does it ten minutes later.”*

Overall, based on the mother’s account, we did not identify any serious issues. Student 3-1 performs reasonably well in math, is weaker in Chinese, especially in written expression, and appears to maintain good peer relationships and a close bond with her sister. Her mother mentioned that as the child grows older, she sometimes says things the mother has never heard or understood before, which makes her feel anxious. Aside from that, the father works as a driver, leaving early and returning late, so he spends little time with the child.
