# Supplementary material for: Psychological wellbeing and social emotional competence of Chinese children and adolescents in the post-pandemic era: patterns, determinants, and interrelations
Source: Front Public Health. 2025 Nov 18;13:1677632. doi: 10.3389/fpubh.2025.1677632 (PMC12668964; doi:10.3389/fpubh.2025.1677632)
Supplement: Supplementary file 4 [file Data_Sheet_4.zip › Supplementary Material 4/Student 3-2_Anonymized.docx]

# Interview Notes: Student 3-2

Student Name: Student 3-2
Grade: Third Grade
the interviewers: the teacher 1, the interviewer, the interviewer, and the the teacher

Student 3-2’s family lives in a newly developed residential community with good overall construction and a pleasant environment. Upon entering the home, we noticed that the desk in the living room was filled with a variety of study materials and books.

*According to the the teacher, Student 3-2 is generally obedient but tends to be playful. The father also mentioned that his son often takes the initiative to share interesting stories and events from school. The child transferred to this school last year and initially had some difficulty adjusting but gradually became accustomed to the new environment. The father emphasized that “good academic performance is important for future employment.” The mother regularly helps him with his studies, printing practice papers and designing exercises to strengthen his learning.*

*The student himself shared that he used to serve as a class monitor at his previous school but has not taken on such a role here, saying, “Maybe everyone here is too outstanding.” He also mentioned that he has started practicing calligraphy and has enrolled in a calligraphy class because he felt his handwriting was not good enough.*

*The parents described him as clever, quick to understand, and persistent. They said he has strong hands-on abilities and great perseverance — “He won’t even eat until he finishes his puzzle.” However, they also noted that he can be a bit emotionally sensitive and tends to cry when criticized.*

the interviewer’s reflection:Student 3-2 appears to be self-disciplined in his studies, and his parents have a well-structured approach to education, providing consistent guidance and supervision. Overall, the family atmosphere, learning environment, and social interactions seem healthy and positive. The previously reported low emotional well-being score on the questionnaire may have been a temporary reflection of the child’s adjustment period after transferring to a new school.
