# Supplementary material for: Psychological wellbeing and social emotional competence of Chinese children and adolescents in the post-pandemic era: patterns, determinants, and interrelations
Source: Front Public Health. 2025 Nov 18;13:1677632. doi: 10.3389/fpubh.2025.1677632 (PMC12668964; doi:10.3389/fpubh.2025.1677632)
Supplement: Supplementary file 4 [file Data_Sheet_4.zip › Supplementary Material 4/Student 3-5_Anonymized.docx]

# Interview Notes: Student 3-5

Student Name: Student 3-5
Grade: Seventh Grade
Date: July 2, 2023
Accompanying Staff: the interviewer, the teacher 1, Homeroom the teacher

Home Visit Record

*Around 11 a.m., we arrived at Student 3-5’s home. The family lives in a village close to the school, which can be considered a kind of “urban village.” the interviewere first entered the basement, where her father works with various car repair tools—he appears to be engaged in automobile maintenance. Both parents were very warm and welcoming, and there was also a younger brother, about three years old. After briefly explaining the purpose of our visit, I went upstairs with Student 3-5 to their apartment on the fifth floor. The home was spacious and bright, and from the living room, one could see a wide expanse of green fields stretching into the distance.*

the interviewere sat on the sofa and began to chat. Student 3-5 said that her academic performance was not very good—she is around the lower middle range in her class. She struggles especially with English and failed her geography exam. She mentioned having one close friend with a complementary personality, and it was clear from her description that their friendship is genuine.

*Student 3-5 is talented in dance and has studied Chinese classical dance for seven to eight years, completing all grading exams. She is currently learning to play the hand drum with a school club and plans to take up street dance and jazz in the future. the interviewerhen talking about her talents, she became visibly more open and animated. Describing her personality, she called herself “carefree and easygoing.” She also enjoys singing, though she admits being a bit tone-deaf. Recently, however, she has set singing aside to focus on improving her grades.*

*She also shared an incident from May, when a “so-called friend” spread rumors and caused conflict with another girl, who even threatened to hit her. Student 3-5 immediately told her parents and teacher, and the matter was quickly resolved. The teacher had the other student write a self-reflection, and peace was restored. Her parents reminded her to stay away from classmates who like to stir up trouble. I found it commendable that Student 3-5 knew to seek help rather than handling the situation alone.*

*She recalled that back in March, she often felt anxious because her grades wouldn’t improve no matter how hard she thought about it. She would lie awake at night wondering why she couldn’t study effectively, but she didn’t take any concrete action. In May, after a heartfelt conversation with her father, things began to change. Her father told her, “It’s okay if your grades don’t improve right away—as long as you’ve tried your best. Nobody is perfect.” That talk helped her relax and shift her mindset. She said she has since become more cheerful and regained her confidence in learning.*

Her father mentioned that he used to rely on harsh discipline—he once smashed two of her tablets and a phone, thinking that removing distractions would make her focus. However, that approach proved ineffective, so he changed his strategy and started communicating more openly with her. This new approach has worked much better.

*Student 3-5 said that during the summer break, she plans to take some time to relax before previewing her eighth-grade materials and gradually improving her grades. She told me, “My classmates also encourage me. I’m now aiming for 300 points and trying to get into No. 3 Middle School.” Although she doesn’t have a long-term plan yet, her short-term goals are clear and achievable, giving a sense of steady progress.*

Finally, Student 3-5 mentioned that her the teacher once called her and said she might have psychological problems, which left her quite confused. This might have been based on her low score from a March survey, but the teacher’s wording was somewhat inappropriate. Fortunately, she did not take it to heart and seems to have regained emotional stability and motivation for learning.

In summary, Student 3-5 experienced emotional distress around March due to academic pressure, but after open communication with her father, she has gradually recovered. She is now more positive and self-aware, steadily moving toward a healthier and more optimistic state of mind.
