# Supplementary material for: Psychological wellbeing and social emotional competence of Chinese children and adolescents in the post-pandemic era: patterns, determinants, and interrelations
Source: Front Public Health. 2025 Nov 18;13:1677632. doi: 10.3389/fpubh.2025.1677632 (PMC12668964; doi:10.3389/fpubh.2025.1677632)
Supplement: Supplementary file 4 [file Data_Sheet_4.zip › Supplementary Material 4/Student 3-6_Anonymized.docx]

# Interview Notes: Student 3-6

Student Name: Student 3-6
Grade: Grade 5
the interviewers: the teacher 1, the interviewer, the interviewer, and accompanying the teacher

It took about ten minutes by car to travel from the county town to a village where Student 3-6 lives. The road was narrow, allowing only one vehicle to pass at a time. There were few houses around the village. Upon arrival at the student’s home, we noticed a small lake right outside and several chickens in the yard. The house looked old and somewhat run-down, with visible signs of age. In the first-floor living room, there was a table and some clothes hanging to dry. The house had two bedrooms: one had been slightly renovated with tiled floors, and family members would take off their shoes before entering. The other room remained rather old and unrefurbished.

Family Background

Student 3-6 is not an only child and has a four-year-old younger sister. According to the mother, the student often helps take care of her sister when the parents are away. The family consists of the grandparents, parents, the student, and her sister, as well as an older cousin with whom the student shares a bedroom. The family lives in a village near a rural county in central China and faces financial difficulties. The home is far from school, so the student must wake up very early—around 5 or 6 a.m.—to take a bus to school.

Relationships and Social Life

The mother said that the student often talks about her school life at home and appears quite cheerful. (This contrasts somewhat with the teacher’s description, suggesting that the student might be somewhat introverted overall.)

Toward the end of the interview, the student mentioned going to the movies with a classmate. The parents of both children communicated to arrange the meeting, which suggests that the two families are familiar and the children share a close friendship.

*The relationship between the student and her father seems tense. The mother explained that the father often criticizes the child—for example, if she scores over 90, he might say, “You must have copied.” The mother described her own parenting style as more like a friendship with her daughter, but she added that “in a family, someone has to be strict,” indicating she does not perceive the father’s harshness as problematic.*

Academic Performance

*According to the the teacher, Student 3-6 is a reliable and disciplined student who performs very well academically. She serves as the class learning monitor, demonstrates strong self-control, and always follows instructions. “She’s one of the students I worry least about,” said the teacher. “So I was quite surprised when I saw her emotional well-being score was very low.” The teacher also noted that because the student is so independent, she might not receive as much individual attention as others.*

*The mother described their approach to education as relatively hands-off, saying, “the interviewere don’t pressure her much, but she must finish her homework.” The student sometimes uses a mobile phone to look up questions she doesn’t understand. Since the mother feels the school’s English curriculum is limited, she has also enrolled her daughter in an English tutoring class.*

Learning Support

Most of the student’s learning support comes from online resources, which she looks up independently.

Personality and Long-Term Development

*The the teacher commented that the student “isn’t particularly outgoing but has her own circle of friends.”*

*The mother described her daughter as cheerful, self-disciplined, and largely self-sufficient since the third grade. Compared with other children, she feels quite satisfied with her daughter’s progress. The student used to take dance lessons but had to stop due to financial constraints. Regarding her hopes for the future, the mother said, “Learning is important—good grades matter, but so does being a good person.”*

the interviewer’s Notes:

*After interviewing the mother and the child, the mother kindly offered me some watermelon. the interviewerhen I finished eating, she said, “Just throw the peel on the ground outside—the chickens will eat it.” For a moment, I wasn’t sure what to do and instinctively looked around for a trash bin.*

*Three impressions from the visit stood out most clearly. First, the overall living environment was quite poor. During the interview, chickens occasionally “flew” into the room, interrupting the conversation. Second, the father’s harsh, critical approach seems to have a noticeable impact on the child’s confidence. Finally, there was the grandmother—she appeared to have difficulty walking and sat quietly throughout the interview, saying nothing, except when she occasionally scolded the younger sister quite sternly. It made me wonder whether she might be equally strict with Student 3-6.At the beginning, the mother seemed dismissive of the child’s low emotional well-being scores, but when I explained some of the specific questionnaire items, her expression became more serious. Overall, I felt that the family does not give enough attention to Student 3-6. This may partly be due to the presence of a second child and a general lack of positive or encouraging parenting practices.*
