# Supplementary material for: Psychological wellbeing and social emotional competence of Chinese children and adolescents in the post-pandemic era: patterns, determinants, and interrelations
Source: Front Public Health. 2025 Nov 18;13:1677632. doi: 10.3389/fpubh.2025.1677632 (PMC12668964; doi:10.3389/fpubh.2025.1677632)
Supplement: Supplementary file 4 [file Data_Sheet_4.zip › Supplementary Material 4/Student 4-3_Anonymized.docx]

# Interview Notes: Student 4-3

Interview Notes: Student 4-3

Home Visit: student4-3

Date: July 2, 2023
Time: 10:00–11:00 a.m.
Participants: Luo Heng, the interviewer the interviewer

*On the way to Student 4-3’s home, the interviewer the interviewer mentioned that he is a very smart but also rather mischievous boy. His academic performance in class is above average but fluctuates a lot. “If he could just devote 5% more effort to studying, his scores would be much higher,” said the interviewer the interviewer. He also described Student 4-3 as mischievous — someone who often refuses to admit mistakes unless the evidence is shown on surveillance footage [so I think what this child says may not always be entirely reliable]. After chatting for a while, we arrived at the apartment complex where Student 4-3’s family rents an apartment. The neighborhood is in the city center, nicely decorated, and well maintained. the interviewer the interviewer said Student 4-3’s father runs a successful business and that their family is relatively well-off. Student 4-3’s mother valued this home visit very much and came downstairs to greet us in person. After entering the apartment, we chatted with his mother for a while. Student 4-3 stayed in his bedroom and did not come out to greet us on his own. His mother admitted that Student 4-3 can indeed be mischievous and rarely listens to her. He seems to fear his father more, who used to discipline him harshly — even physically — but now that Student 4-3 is older and in his rebellious phase, his father no longer hits him. She also mentioned that Student 4-3 had been in a nearby city, a nearby city, the previous day and had to be called back home for this interview, which made him unwilling and caused an argument. After a few polite exchanges, we asked his mother to call him out of his room due to time constraints.*

*My first impression of Student 4-3 was that of a “cool” boy — his hair dyed purple, wearing a T-shirt featuring Marilyn Manson, the American heavy metal rock legend. At first, I thought he would be defiant and difficult to talk to. However, that concern quickly disappeared. Student 4-3 was actually quite engaged in the conversation and answered questions seriously and politely throughout. When we discussed his low scores on some of the questionnaire items, he admitted that they reflected his emotional state at the time. He said he was feeling down and lonely back then — he had few close friends and often argued with teachers — so he had rated himself very low. “But now I’m doing much better,” he said. When asked what had changed, he said he recently did a few “meaningful, positive things,” such as helping an elderly woman recover her money from a telecom scam and helping younger students resist bullying from upperclassmen [these stories sounded a bit dramatic, so I’m not sure if he made them up on the spot to please me]. Student 4-3 also admitted that he sometimes struggles to control his temper and can suddenly explode with anger [the interviewer the interviewer later told me that his father also has a bad temper, suggesting a possible genetic link]. When asked what usually triggers his anger, Student 4-3 mentioned three things. First, when his parents don’t listen carefully to him — he feels they fail to communicate properly. “It’s like when I ask whether the water is hot or cold, they’ll just tell me it’s sugar water and then lecture me that sugar water is bad for my health.” Second, being wrongly accused. He shared an incident in his math class: he had put an ice pop in his cup intending to let it melt into a drink, but his teacher insisted that he had eaten it. When the teacher refused to believe his explanation, he became very agitated. Third, broken promises. He said that once his mother agreed he could invite a friend over to play in his room and promised not to disturb them, but when the friend came, she repeatedly entered the room to “check” on them. This upset him greatly, even though his friend tried to reassure him that it was no big deal. Although he often quarrels with his parents, Student 4-3 said he never stays angry for long. “Usually we just argue for a bit, and then I’m the one who gives in first — I joke around, make them laugh, and things go back to normal. I know my parents mean well, so there’s no point in keeping silent or holding grudges. People just need a way to back down gracefully,” he said. [This shows that Student 4-3 is actually someone who weighs pros and cons, knows when to compromise, and is willing to resolve conflicts. His anger seems to be momentary — he can regulate his emotions and calm down quickly.]*

*One thing that left a deep impression on me was that Student 4-3 is actually quite nostalgic and doesn’t adapt easily to new environments. After finishing primary school in a nearby city Town, he moved to study at a local middle school in a local county. Although he has been there for two years, he still misses his friends from a nearby city. He enjoys spending time with them during the holidays. When I asked whether he would like to study abroad one day, he shook his head firmly, saying that he wouldn’t adapt to life overseas and preferred to stay in China [this answer surprised me — with his dyed hair and love for rock music, I thought he might long for the “freedom” of life abroad. But in essence, he’s still a sentimental boy who values familiarity and comfort]. However, he added that he would like to attend high school in a city in central China to experience something different. When asked about his hobbies, he said that his interests tend to fade quickly — for example, he used to love playing basketball but suddenly lost interest, feeling it was meaningless. The same thing happened with gaming — he simply stopped one day. So, he has now set himself a goal: to study hard in the third year of junior high and see how far he can go if he truly gives it his all. [the interviewer the interviewer later sent me a photo of Student 4-3’s bedroom wall, covered with motivational notes like “Get into a 985 university,” “Peking University,” and “University of Electronic Science and Technology of China.”]*

In my view, Student 4-3 is a typical adolescent boy — rebellious on the outside, but still craving friendship and approval deep down. His conflicts with his parents are within normal bounds. Though he occasionally loses control of his emotions, he is able to self-regulate and resolve conflicts soon after. At present, he may lack clear goals and a strong sense of purpose, but as he invests more energy into studying and exam preparation, many of his troubles are likely to fade away.
