# Supplementary material for: Psychological wellbeing and social emotional competence of Chinese children and adolescents in the post-pandemic era: patterns, determinants, and interrelations
Source: Front Public Health. 2025 Nov 18;13:1677632. doi: 10.3389/fpubh.2025.1677632 (PMC12668964; doi:10.3389/fpubh.2025.1677632)
Supplement: Supplementary file 4 [file Data_Sheet_4.zip › Supplementary Material 4/Student 4-4_Anonymized.docx]

# Interview Notes: Student 4-4

Interview Notes: Student 4-4

Home Visit: student4-4

Date: July 2, 2023
Time: 9:30–10:30 a.m.
Participants: the interviewer, the interviewer (Homeroom and English the interviewer)

At nine in the morning, the interviewer the interviewer and I departed from a local middle school and drove to Student 4-4’s home. It seemed that her residence was located in a rather remote area. the interviewer the interviewer, who was quite skilled at using his voice assistant, said: 'Call the twins.' After several phone calls with Student 4-4’s mother, we finally confirmed the correct location [Compared with the relationship between primary school teachers and parents, middle school teachers seem to be much more familiar with the parents. Based on the interviewer the interviewer’s contact note labeled 'the twins,' I guessed that the 'brother' mentioned in Student 4-4’s questionnaire was her twin brother].

Student 4-4’s family lives in an old, traditional residential complex without an elevator. Her mother warmly greeted us at the door. Also present were Student 4-4’s aunt (who works in a southern city in China and occasionally returns home), grandmother, and grandfather (who was resting in the bedroom but later came out to meet us). The apartment was fairly spacious — about three bedrooms and one living room — and kept very tidy. Student 4-4’s mother had even prepared fruit for us.

Through conversation with her mother, I learned that Student 4-4 communicates fairly often with her family. Her mother is familiar with several of Student 4-4’s close friends. However, due to past academic struggles, Student 4-4 had lost confidence, becoming introverted and reluctant to go out, shutting herself off from others. Her mother said that she has improved a lot recently and is now much more cheerful. She added that she doesn’t demand high grades and cares more about Student 4-4’s physical and mental well-being. Student 4-4’s aunt agreed, saying that 'society is so competitive nowadays — as long as she has basic survival skills, that’s enough. We don’t want to put too much pressure on her; she should learn at her own pace.' In addition, Student 4-4’s mother said that Student 4-4 enjoys playing the piano and hopes she will continue with this hobby. Later, during my conversation with Student 4-4, she told me she plans to go to a southern city in China in August to learn the violin. Perhaps her mother hopes to cultivate a particular artistic skill in her. When asked about Student 4-4’s strengths, both her teacher and mother agreed that she is very responsible and completes any task assigned to her with great care. From this conversation, I could clearly sense that her mother and aunt care deeply about her and do not hold a 'grades-only' mindset [although it is also possible that this was something her mother prepared in advance, knowing that the teacher would be visiting]. Still, I wondered — why did Student 4-4 previously close herself off so severely? With this question in mind, I began a private conversation with her.

At the beginning of our chat, Student 4-4 immediately told me that most of her stress actually comes from her brother. 'When I started seventh grade,' she said, 'some of my brother’s friends would ask why, if we share the same parents, his grades are so good while mine are so poor.' This deeply upset her and was the main reason for her depression and reluctance to communicate with others. the interviewerreover, when classmates 'teased' her, her brother always sided with his friends.

In addition to teasing her at school — once, after Student 4-4 used a whitening sunscreen, her brother and some boys joked that she looked like a 'gangster,' which made her very upset — he also fought with her at home. Fortunately, their parents always took Student 4-4’s side and scolded her brother together. She said that although her father works in a southern city in China, whenever she tells him about her brother hitting her, he always lectures him when he comes home for holidays. [I thought, luckily, her parents do not hold traditional son-preference attitudes.]

All these experiences combined made Student 4-4 feel extremely insecure and unconfident, unwilling even to speak with others. 'Sometimes,' she said, 'I didn’t even want to talk to my mom. When my parents asked me questions, I just ignored them.'

Student 4-4 said, 'I’m much better now, because I have a very, very good friend at school. She laughs a lot, and her laughter is contagious.' Influenced by this friend, Student 4-4 said she has gradually become less withdrawn and is changing for the better. For example, when her brother turned her photo into a meme and shared it in a group chat, she felt unhappy but tried not to let it affect her mood. I believe her — she truly seems to be coming out of her shell.

During our talk, Student 4-4 also shared stories about her classmates. Like many students her age, she talked about the 'gossip' between boys and girls at school — how boys like to discuss girls, watch pornographic websites, and who’s dating whom [in fact, such gossiping may symbolize Student 4-4’s re-engagement in social interaction — a positive sign compared to her previous self-isolation]. I thought to myself, 'After all these years, the same scenes from our own middle school days are still playing out among them — perhaps this is part of the uniquely Chinese adolescent experience.'

Near the end of our conversation, perhaps feeling closer to me, Student 4-4 revealed that her mother had taken her to see a psychologist some time ago. The psychologist told her that even though her brother is family, if he makes her unhappy, she can choose not to listen to him. If she ever feels upset again, she can go to the hospital and talk to the psychologist. Upon hearing this, I realized — this must be the key reason Student 4-4 has become more cheerful [though I’m not sure whether her 'cheerful friend' actually exists — perhaps this 'laughing friend' is the psychologist herself? Maybe I should have asked a bit more….

When asked about her future goals, Student 4-4 said she wants to study IT and become a hacker because she thinks it’s cool. Her uncle also buys her programming books to read.

In conclusion, Student 4-4 is a careful, sensitive, yet fundamentally cheerful girl who once suffered from deep insecurity due to her brother’s behavior and other factors. Everyone blooms at their own pace — there’s no need to compare with others. She only needs to focus on being herself, and her time to blossom will surely come.
