# Supplementary material for: Psychological wellbeing and social emotional competence of Chinese children and adolescents in the post-pandemic era: patterns, determinants, and interrelations
Source: Front Public Health. 2025 Nov 18;13:1677632. doi: 10.3389/fpubh.2025.1677632 (PMC12668964; doi:10.3389/fpubh.2025.1677632)
Supplement: Supplementary file 4 [file Data_Sheet_4.zip › Supplementary Material 4/Student 4-5_Anonymized.docx]

# Interview Notes: Student 4-5

Interview Notes: Student 4-5

Home Visit: student 4-5

Student Name: Student 4-5 Grade: Seventh Grade
Date: July 2, 2023
Accompanying Staff: the interviewer, Director the interviewer, Homeroom the interviewer

*At around ten in the morning, our group arrived at Student 4-5’s home. The family lives in an old residential neighborhood located within a small alley. Student 4-5 and her mother had been waiting for us at the door under umbrellas. Although their home is not very large, it feels cozy. The living room walls are covered with many award certificates and her calligraphy works. There are also wedding photos of her parents and a family photo of four people. However, later I learned from Student 4-5 that the “brother” in the photo is actually her cousin from her aunt’s family — she is in fact an only child.*

*Student 4-5 is rather introverted and speaks softly. Her homeroom teacher described her as “an average student, performing at a medium level academically but with high expectations for herself.” In junior high school, her average score is around 80–90 points (out of 120), while in primary school her grades were higher — in the 90s (out of 100). Student 4-5 said that her weaknesses lie in English sentence structures and vocabulary: “I can hear them, but I don’t understand.” She finds “pursuit problems” in math challenging, and for subjects like Politics, History, Geography, and Biology, she often struggles when question types change and doesn’t know how to approach them. It can be seen that Student 4-5 is aware of her academic shortcomings, but her learning methods may lack flexibility — perhaps explaining why her grades have plateaued.*

*Student 4-5 has made an online acquaintance through gaming — someone about her age. According to her, this friend is one year older and often ranks among the top five in school. They have exchanged contact information and occasionally talk about schoolwork and personal frustrations. (I suspect that Student 4-5 may not wish to share her true thoughts with people around her, but she occasionally communicates with nearby friends.) However, she does not confide much in her family. Student 4-5 said, “My impatience comes from my family. Whatever I do, they accuse me of playing on my phone. I can’t explain myself. Whatever I do, my mom questions me — sometimes she yells, glares at me, throws things. Just because I didn’t do my homework, didn’t mop the floor, or skipped chores…” It is clear that communication between Student 4-5 and her mother is limited and problematic.*

*When asked if she had anything that made her happy, Student 4-5 replied, “No.” But when asked what made her unhappy, she said, “My mom often loses her temper with me. Sometimes I stay upset for a whole week.” When her mom gets angry, she tries to appease her by sending WeChat messages or red envelopes. Recently, her mom scolded her for not cleaning the house properly before our visit. When asked about her strengths, Student 4-5 said she couldn’t think of any, then softly asked, “Does being good at chores count?” Student 4-5 admires soldiers and the strong, agile characters in games. In the future, she hopes to travel across China like her aunt — and her dream destination is Australia.*

One event that impressed me deeply was that Student 4-5 experienced school bullying in sixth grade. Nearly ten peers verbally and physically bullied her. The incident began when a girl confessed her affection to Student 4-5, but she refused and blocked her on social media. Afterward, the bullies often rang the doorbell downstairs at Student 4-5’s home. Later, during her transition to middle school, she deliberately chose a local middle school to avoid them after hearing that they enrolled in a local middle school. During the bullying, Student 4-5 never sought help from teachers or her parents. She said she didn’t tell teachers because she feared they would inform her parents. This shows that Student 4-5 does not fully trust her parents.

*Student 4-5 once tried to indirectly ask her mother about homosexuality but was met with the response, “You’re sick.” She also once tested her father’s attitude through DingTalk, asking, “If a dozen people bully one person, does that count as bullying?” Her father replied, “That’s the problem of that person.” Perhaps seeing such reactions from her parents made Student 4-5 believe that even if she spoke up, they wouldn’t side with her — so she endured everything silently. When asked if she often feels unhappy, she said, “Yes, often. My mood changes for no reason. Sometimes even in happy situations, I suddenly lose interest.”*

In conclusion, Student 4-5 is a diligent and thoughtful child who lacks communication with her parents because she feels they wouldn’t understand her. Her mother’s emotional volatility has a strong influence on her. Student 4-5 needs more open communication and guidance, as well as help in both learning strategies and emotional regulation.
