# Supplementary material for: Psychological wellbeing and social emotional competence of Chinese children and adolescents in the post-pandemic era: patterns, determinants, and interrelations
Source: Front Public Health. 2025 Nov 18;13:1677632. doi: 10.3389/fpubh.2025.1677632 (PMC12668964; doi:10.3389/fpubh.2025.1677632)
Supplement: Supplementary file 4 [file Data_Sheet_4.zip › Supplementary Material 4/Student 4-6_Anonymized.docx]

# Interview Notes: Student 4-6

Interview Notes: Student 4-6

Interview: student 4-6

Interviewers: the interviewer, the interviewer, the interviewer (Homeroom the interviewer)

On the way to Student 4-6’s home, the interviewer the interviewer reminded me that the next student’s situation might be quite serious — she ranks at the very bottom of the class — and told me to be mentally prepared.

Student 4-6’s family runs a small supermarket. When we arrived, her mother was standing behind the cashier counter, and it was her father who came out to greet us. He warmly offered us bottles of cold mineral water from the refrigerator. Then, he led us to a guesthouse next to the supermarket, where I had a private conversation with Student 4-6 in one of the rooms.

*“I often feel that life is meaningless. I cut my arms with a knife. I think the blood looks beautiful…” Before I even began asking questions, Student 4-6 said this and showed me the scars on her left arm, which were clearly made by a knife. During our talk, I learned about her upbringing. Before the age of ten, she lived in the countryside with her grandmother, who made a living by farming vegetables to support her schooling, while her parents worked away from home. “Back then, I was actually good at making friends,” said Student 4-6. Later, her parents, feeling that her grandmother was working too hard, decided to bring her to a local county, where they opened this supermarket. They also transferred her to a local primary school in the fourth grade. That, in my view, seemed to mark a turning point in her life. According to Student 4-6, after transferring schools, she experienced severe bullying. The class monitor led other students in extorting her for money and bullying her. She had once sought help from her parents and teachers, but her father dismissed it, and the teacher chose to believe the class monitor rather than a new transfer student. Things grew worse — the whole class began calling her names, hurling insults, humiliating her, and isolating her. During lunch breaks, several girls would push cleaning tools into her face in the dormitory. One incident escalated when she was shoved by a classmate, leaving deep scars on her face and legs. “I was disfigured,” she said — the scars took two years to heal. After the interview, I consulted a teacher from a local primary school, who vaguely remembered the incident but said he was told that Student 4-6 “fell on her own.”*

*The isolation and bullying did not stop in middle school. “After my elementary school classmates told my nickname to others, my middle school classmates started calling me by it too,” she said. She had again sought help from teachers, but the harassment only paused temporarily — when the teacher was around. Once the teacher left, the bullying resumed as before.*

*When she was studying in the countryside, Student 4-6 had a close friend a few years younger than her. “We were very close — we could talk about anything,” she said. However, that friend later moved to a southwestern province in China, and now they can only communicate via phone.*

*Student 4-6 rarely talks to her parents. She said her parents often wrong her, scold her, and even beat her. “I try to talk to them. I cry and tell them how I feel, but they don’t understand,” she said. “Why do I have to take the blame for someone else’s mistake? Just because he’s my cousin? Just because he’s a guest?” She was referring to an incident where her younger cousin (her uncle’s son) stole a box of trading cards, but her mother blamed her instead, saying it was improper to accuse a guest. Student 4-6 mentioned that she loves drawing and anime and has long wanted to take art classes. However, her parents refused, despite her persistent requests for two years. “My grades are bad, but if I can just score 300 points, I can pursue the art track. I know how to make up that 300 points, but my parents won’t let me join the class.” Recently, with a teacher’s help, her parents nearly agreed — but her mother changed her mind again. Student 4-6 described that she and her parents live in the apartment above the supermarket, all three sharing one room. Her bed is placed in a narrow hallway where her father once installed a clothesline. When she questioned why he installed it directly above her bed, he brushed it off. Once, during an argument, her father smashed her second-hand tablet, which she had bought cheaply. After she got it repaired, it mysteriously disappeared, but her father insisted she had lost it and refused to believe her — trusting her mother instead. Student 4-6 said she knows the family’s financial situation isn’t good. Her father bought a retirement apartment and owes the bank over 500,000 yuan. She often helps deliver bottled water for the store and said, “My dad carries two barrels up to the fifth or sixth floor — it’s really hard work.”*

*Student 4-6 said she likes watching TV dramas and following celebrities. “That’s how I relieve stress — they’re my light, the hope I want to pursue,” she said. She once dreamed of working at her favorite celebrity’s studio, but when she told her family, both her parents and cousin mocked her: “Do you think someone like you could ever get hired there?” She said she felt heartbroken and disappointed — she hadn’t expected her cousin to be among those who ridiculed her.*

*“Sometimes I wonder whether this world would be better without me — whether my parents would be happier without me…” Student 4-6 expressed suicidal thoughts. She said she often loses interest in everything, doesn’t want to eat, and cries for no reason — unable to stop even when she doesn’t want to cry. “the interviewerm thinks it’s because I play with my phone too much.” Drawing is her only interest, but after two years of self-teaching through online videos, she feels she has stopped improving. She thought her drawings are ugly and really want to take a class, but her family just won’t let me.*

Overall, Student 4-6 has experienced severe school bullying, which continues to this day. She has not received understanding or support from her parents or teachers. Communication with her family is minimal and emotionally shallow, leaving her feeling misunderstood and isolated. Her only close friend lives far away, and their digital connection offers little comfort. However, the fact that she willingly spoke so much to me today is, in my view, a cry for help. She still longs for understanding and attention — and dreams of studying art. This should be a key focus of follow-up support in the future.
