# Supplementary material for: Psychological wellbeing and social emotional competence of Chinese children and adolescents in the post-pandemic era: patterns, determinants, and interrelations
Source: Front Public Health. 2025 Nov 18;13:1677632. doi: 10.3389/fpubh.2025.1677632 (PMC12668964; doi:10.3389/fpubh.2025.1677632)
Supplement: Supplementary file 4 [file Data_Sheet_4.zip › Supplementary Material 4/Student 5-2_Anonymized.docx]

# Interview Notes: Student 5-2

Student 5-2

Interviewers: teacher the interviewer and one the interviewer

Date: July 2, 2023 (11:00 a.m. – 12:00 p.m.)

Student 5-2 home is about a ten-minute drive from middle school. However, the surrounding area seemed rather remote, more like a rural village than a county town. She and her family live in a two-story rural house with a small courtyard. She lives with her grandfather, grandmother, father, and stepmother, who recently gave birth to a baby boy.

Student 5-2 appeared very shy and gentle. While the principal chatted with her family, I conducted a one-on-one interview with her in a separate room.

She spoke in a very soft voice—so soft that it was difficult to hear her clearly at first. After I asked her to speak a little louder, it improved slightly. I followed her questionnaire responses and asked about each item in turn.

Regarding loneliness, she said she didn’t have many friends—only one or two close friends—but if they occasionally left her out at lunch, she would feel lonely and sad, especially when seeing other students gathering in groups. [I comforted her by saying that having one or two true friends was already enough, and that eating alone could also be a kind of freedom. Yet I couldn’t help but wonder—are those one or two people truly her close friends? If so, why would they leave her out? Or is it perhaps one-sided on her part?]

When asked about the biggest difficulties in her life and studies, she said her poor academic performance troubled her the most. She was always near the bottom of her class, which made her feel deeply unconfident and confused about the future. Whenever classmates discussed mock exam scores or which high schools they might attend, she felt anxious, knowing that she would probably fail to reach the admission line for any high school. Yet no one had ever explained to her what would happen if she couldn’t enter high school, or what her life might look like afterward.

She said that her grades had been fine in primary school, but had dropped sharply since entering junior high. [I could only encourage her not to give up, telling her that the principal was planning differentiated instruction so that struggling students like her might still have a chance to fill in their gaps. I wasn’t sure whether her parents had taken any measures to help; it seemed that neither her father nor stepmother cared much about her grades—not in a harsh or punitive way, but simply with indifference.]

When the topic turned to her parents’ divorce, she was reluctant to talk, nor did she want to discuss her stepmother or the new baby brother. She said she missed her biological mother very much but didn’t dare to contact her, knowing that her grandparents disliked her mother.

*Because of her academic struggles, she had a very low self-evaluation and felt that she was worthless and incapable of success, that life was meaningless. When she said this, her eyes turned red—it was clear she was in pain. I asked directly, “Have you ever had suicidal thoughts?” As I expected, she nodded and admitted that she had once tried to cut her wrists. She had told no one—not her parents, teachers, nor any counselor—and had not sought psychological help. Her family had not noticed any unusual behavior, likely because they had not been paying attention to her.*

Her very soft voice fit the profile of a person with depression, so I asked further questions. She reported insomnia, often unable to fall asleep until one or two in the morning, as well as frequent dizziness and stomach pain—all symptoms consistent with depression. [She said these problems began in her first year of junior high school. I suspect that her declining cognitive functioning due to depression led to her drop in academic performance, which in turn worsened her depressive symptoms—a vicious cycle. As for the trigger, it was likely the parental divorce she preferred not to discuss.]

Student 5-2 displayed clear suicidal ideation and had attempted self-harm, requiring urgent attention and professional intervention, including both medication and psychological counseling.
