# Supplementary material for: Psychological wellbeing and social emotional competence of Chinese children and adolescents in the post-pandemic era: patterns, determinants, and interrelations
Source: Front Public Health. 2025 Nov 18;13:1677632. doi: 10.3389/fpubh.2025.1677632 (PMC12668964; doi:10.3389/fpubh.2025.1677632)
Supplement: Supplementary file 4 [file Data_Sheet_4.zip › Supplementary Material 4/Student 5-3_Anonymized.docx]

# Interview Notes: Student 5-3

Student 5-3

Interviewers: one teacher from middle school, one the interviewer
Date: July 2, 2023, 8:30–10:00 a.m.

The teacher drove me to Student 5-3’s apartment, which was hidden behind a row of small neighborhood shops. We followed a resident through the gate, as entry required an access card. Student 5-3’s home was on the 19th floor. When we arrived, the door was already open, and both Student 5-3 and his grandmother were waiting for us in the living room. After we entered, the grandmother offered us chairs and poured some tea. The teacher explained that the homeroom teacher could not come due to an emergency, but the grandmother responded warmly that it didn’t matter—whoever came, they were there to care about Student 5-3.

After I gave Student 5-3 a small gift, I suggested taking a photo together. The grandmother eagerly joined us, clearly pleased by our visit. I began chatting with her to understand the family situation.

According to the grandmother, Student 5-3’s family circumstances were very special. His father had passed away, and his mother had left soon after. The household expenses were mostly supported by Student 5-3’s aunt, who occasionally complained about the financial burden. For a few years, the grandmother refrained from asking her for money, worried about becoming a burden since the aunt had her own family to care for. The grandmother said Student 5-3 was very obedient because he feared that she might abandon him.

Throughout our conversation, Student 5-3 sat quietly on the sofa reading a book—but I noticed he wasn’t turning the pages, clearly listening to us. When the grandmother mentioned the father’s death and the mother’s departure, he began to cry. (The grandmother did not avoid mentioning these painful facts in front of the child. Although she wept as she spoke, it seemed she had already accepted the situation. However, Student 5-3 was still young, and each reminder made him cry again; it would be better not to repeat these things in his presence.)

*The grandmother described Student 5-3 as well-behaved. He usually stayed home reading and writing. She mentioned that she planned to take him back to the countryside soon, where they could grow vegetables and collect firewood to save on living expenses. She said she feared strangers knocking on the door and rarely told neighbors the truth about their family situation, believing that an old woman and a child living alone was unsafe. (Indeed, it was clear that her situation was extremely difficult, both financially and emotionally. Although the aunt sometimes complained about providing money, she still encouraged the grandmother to hold on—“Once he grows up, things will get better.” That encouragement seemed to be her main source of strength.)*

Because the grandmother spoke in dialect and I could not fully understand her, I decided to talk privately with Student 5-3. He agreed and led me into his bedroom, helping me move a chair and close the door.

*During our conversation, he said he often felt anxious and conflicted. His mother had wanted to take him to a southern city in China for a visit, but he couldn’t go—he said it was because “low-income households aren’t allowed to contact their mothers.” (Neither he nor I understood what that meant; it was probably an explanation his grandmother had given him.) The fact that he finally had a chance to see his mother, only to be denied it again, was what made him saddest.*

Student 5-3 also felt family pressure. Sometimes, when he daydreamed in class, he imagined how nice it would be if his father could come back—it would fill the missing void of paternal love. (At this point, he burst into tears. I handed him a tissue and gently patted his shoulder.)

He told me he sometimes secretly called his mother using his smartwatch because his grandmother disliked him contacting her. The grandmother believed that his father had died after arguing with his mother and therefore forbade all contact. (I believe this was deeply harmful—he had already suffered the trauma of losing his father, and now he was being denied the only remaining maternal connection. For such a young child, this burden was heartbreaking.)

At school, he had few friends. He said that reading was his main pastime—but not because he enjoyed it; rather, it helped distract him from thinking about painful things. He mentioned that he got along better with younger children from his neighborhood. (This contrast suggests that his classmates probably knew about his family situation, making him feel inferior and withdrawn, whereas the neighborhood children did not, allowing him to relax and play freely.)

When asked about his strengths and weaknesses, Student 5-3 thought for a long time and said he had no strengths. He said his weaknesses were that he cried easily, wasn’t good at expressing himself, and felt nervous around strangers. I encouraged him, saying he was expressing himself well now and speaking clearly. He smiled and said he didn’t feel nervous talking to me. I told him that when guests visit, it’s polite to greet them first; if he didn’t want to talk, he could excuse himself by saying he needed to study, but he shouldn’t leave without saying a word. (As he spoke, I thought of my own childhood—often scolded for being quiet—and realized that no one might have taught him these basic social norms, so I offered some simple guidance.)

Student 5-3 said he was not satisfied with his life, feeling under great pressure and struggling academically. However, he was happy about the summer vacation, because he disliked the crowded school environment and finally had a quiet space to think. I told him that every day passes anyway, so he should try to think about pleasant things—like whom to play with after school or what to eat for dinner—rather than dwell on worries.

When asked about his interests, He said he liked drawing, roller-skating, and riding his bicycle. Unfortunately, his roller skates were broken, and he looked disappointed as he said this. (Given his family’s financial situation, it was clear he couldn’t easily get what he wanted. The longing for something he loved but couldn’t have might turn into an emotional fixation.)

As our conversation continued, his mood gradually brightened. He said he believed his life would get better and that if he couldn’t go to a southern city in China this year, going next year would be fine too. (Throughout the interview, I paid close attention to his emotions, trying not to touch sensitive topics. The room was quite warm, so I fanned him occasionally while he talked. He said he felt happy chatting with me, and I could see his mood lift. When I finally left, he walked me to the door and said goodbye loudly.)
