# Supplementary material for: Psychological wellbeing and social emotional competence of Chinese children and adolescents in the post-pandemic era: patterns, determinants, and interrelations
Source: Front Public Health. 2025 Nov 18;13:1677632. doi: 10.3389/fpubh.2025.1677632 (PMC12668964; doi:10.3389/fpubh.2025.1677632)
Supplement: Supplementary file 4 [file Data_Sheet_4.zip › Supplementary Material 4/Student 5-6_Anonymized.docx]

# Interview Notes: Student 5-6

Student 5-6

Interviewers: one school teacher and one the interviewer
Date: July 2, 2023

*Student 5-6 lives on the sixth floor. In the living room, there were three square tables—two clearly mahjong tables, and the third, larger and taller, stacked with books and papers. He said that this was where he usually did his homework. His grandmother greeted us at the door, and both he and his half-brother came out to welcome us. Student 5-6 was wearing a sleeveless white shirt, revealing well-toned arms. After some small talk, I asked to speak with Student 5-6 privately. He led me into his grandmother’s bedroom. When I asked if we could go to his own room, he said, “There’s nothing to see there. Let’s just stay here.”*

Through the conversation, I learned that his family background was very complex. Before third grade, he attended school in a local county County, cared for by his grandmother and her second husband, while his biological parents worked in a southern city in China. Later, his parents divorced, and his mother moved to a metropolitan city in eastern China to live with her new husband—his current stepfather. Student 5-6 also transferred to a school in a metropolitan city in eastern China. Because he was born in September, he was too young to enroll in a public fourth grade that year, so he first attended a private school, repeating fourth grade. During that year, a physical education teacher recognized his athletic potential and encouraged him to develop his sports talent. However, his academic performance, previously strong in a local county, declined sharply, and he began to lose confidence in his studies. As his grades worsened, he eventually transferred back to a local middle school in a local county during seventh grade.

*When we talked about his fitness routine, he became animated. From Monday to Friday, he trained his shoulders, abs, and arms; weekends were his “cheat days,” when he allowed himself some snacks. He insisted on two hours of training every day. When I asked if he trained out of passion or enjoyment, he quickly shook his head. In his view, fitness and sports were not hobbies but his future career path. Since his grades were poor, he believed he must train hard and compete to earn money and achieve the ideal life he envisioned.*

*He said that Douyin (TikTok) was his window to the world—it broadened his horizons and connected him with “many like-minded people,” including those he called “world-class athletes.” He had joined an online community where they chatted and shared workout experiences. He also ran his own Douyin account, setting two major goals: to grow his online presence and to complete his current fitness plan. He had set a personal target: to finish his training program before age fourteen and then enroll in a sports academy to compete in physique contests.*

*In real life, Student 5-6 admitted he had almost no friends. Although he played with some classmates, he didn’t consider them real friends. In his words, “Friends are people who share the same goals and can help each other.” He believed that classmates were temporary—“they just pass through your life for a while and won’t stay forever.”*

*He viewed studying as a burden, something that interfered with his workout schedule. It irritated him to spend time on homework instead of training. Still, he planned to study abroad in the future for professional athletic training, acknowledging that English might be important. Yet, despite recognizing this, he took no action, saying, “Once I go abroad, being in an English environment will naturally improve it within a year or two.”*

When asked about his biological father, he said he had almost no memory of him—just a vague impression of his face—but added that it didn’t matter and had no impact on him. Throughout our conversation, whether discussing relationships, family, or his grandmother who raised him, Student 5-6 appeared emotionally detached. He said he was grateful to those who raised him and would repay them in the future, but he didn’t feel any particular emotional attachment.

*When asked how he handled stress, he said he would cry or simply wait for it to fade. For example, he wanted to attend a sports school, but his father disagreed. After much persuasion failed, they had a serious argument the previous night, and he cried. “Crying helps me release emotions,” he said, “but it doesn’t solve the problem.”*

Overall, Student 5-6 demonstrated clear goals and discipline in pursuing them. Although he seemed emotionally distant in interpersonal relationships, this detachment had not yet led to negative outcomes. He was highly self-directed, with a strong sense of purpose and personal agency.
