# Supplementary material for: Psychological wellbeing and social emotional competence of Chinese children and adolescents in the post-pandemic era: patterns, determinants, and interrelations
Source: Front Public Health. 2025 Nov 18;13:1677632. doi: 10.3389/fpubh.2025.1677632 (PMC12668964; doi:10.3389/fpubh.2025.1677632)
Supplement: Supplementary file 4 [file Data_Sheet_4.zip › Supplementary Material 4/Student_2-1_Anonymized.docx]

# Interview Notes: Student 2-1

After the first interview, we planned to visit Student 2-1's home. However, the contact information was incomplete, so we had to reach out to others before confirming the address. After contacting the parents, we set off.

After knocking on the door, a woman opened it. It was Student 2-1's mother, who warmly invited us in. On the short walk from the front door to the living room stood a little boy, about one year old, licking a cold chocolate popsicle fresh from the refrigerator. He smiled as he looked at me, his eyes narrowed. This was Student 2-1's younger brother. Student 2-1 wasn't home, and her mother told us that her grandmother had taken her to visit her great-grandfather, who is now in his eighties.

*Student 2-1's mother is very talkative. She explained that her daughter's grades dropped on this year's midterm exam. She didn't criticize her, simply saying, "You have one good thing: at least you didn't cheat, so your grades are real." She then bought her books and materials for concentration training, and every night after finishing her homework, she practiced visual, auditory, and mathematical exercises. Her math score on the midterm was only over 40, but it rose to over 80 on the final. "I'm very pleased that she's been able to persist in this concentration training every night for over two months," her mother said. "She often asks me, 'Mom, do I have to study? Are there other options?' I tell her there's more than one path. After the midterm exam, I talked to her about two options. One is to study hard and go to college like my cousin, learning knowledge at a higher level and with a broader perspective. Or you can go to a technical secondary school, learn a trade, and still make a living, just a little harder. You don't have to tell me now, you can think about it and tell me tomorrow.' The next day, she told me she still wanted to go to college. I said, 'Okay, let's work together.'" It's clear that her mother is very dedicated to her child's education and places great emphasis on communication and interaction with her.*

*Student 2-1's mother also took the initiative to discuss the topic of premature love. "One day, she came home and asked me, 'Mom, if I'm in love too early, will you blame me?' When I heard that, my heart was boiling, but I couldn't show it. I asked her, 'What does this boy like about you? Are you the prettiest in the class? Do you have the best grades? If he likes you because you're pretty, he might also like someone else because they're prettier. If he likes you because you have good grades, there must be someone with better grades in the class.' She couldn't answer. I asked her if she knew what dating and marriage were, and she said she didn't know either. I asked her if there were many dating people in the class, and she nodded and said, 'A lot.' After a while, she probably forgot about it herself, and the matter was dropped." On the one hand, this shows that the mother and child communicate frequently, and the child is willing to share these feelings with her. On the other hand, the interpersonal environment in the class may influence other students to blindly yearn for and imitate romance.*

*Student 2-1's mother also mentioned that her father is extremely busy with work and frequently travels around the country, resulting in very little interaction with her daughter. As a result, the daughter is particularly resistant to her father, unwilling to call him “Dad,” and even dislikes him. "He never pays attention to me, and when he comes, he only criticizes me." Student 2-1's mother explained that she now acts more as a communication bridge between father and daughter, encouraging her father to pay more attention to her, asking about her test scores, what happened at school, and allowing her to say hello to him via video. The mother believes that the child lacks courage due to the lack of her father's presence. Before the pandemic, because her father was constantly traveling for business, she would take her daughter on trips around the country every holiday.*

*When asked about her expectations for her daughter, the mother replied that she doesn't expect her to get perfect scores or even get the top score. Of course, not all children are academically gifted, but she simply hopes that she will put her heart and soul into whatever she does, and not just slack off. "We're thinking of saving 200,000 to 300,000 yuan for each of our two children, so they can use it as capital if they want to buy a house or pursue their own interests."*

*I continued to talk with Student 2-1's mother. "In fourth grade, there was a period where she would suddenly get up and sit up in bed. I didn't know why, so I called the teacher. The teacher said, 'Your child can't sleep because she hasn't finished her homework, and it has nothing to do with us teachers.' I was furious and wanted to go to their teacher's office, but I was afraid he'd harass my child, so I didn't go. Later, when I talked to her about it, she told me, 'I finally understand why the teacher kept scolding me in class during that time. It's normal for me to be unable to sleep because I haven't finished my homework. I didn't have any friends during that time.' I think teachers and parents, teachers and students are two different things, but it's clear that my child is being isolated at school," her mother said angrily.*

There's a treadmill on the balcony at home. It's for Student 2-1, and the adults use it occasionally. Student 2-1 may be a bit heavier than her peers. Although she doesn't usually show it, she sometimes deliberately pulls her clothes down while walking and chats with classmates about her outfit. Her mother said she's concerned about her child's weight.
