# Supplementary material for: Psychological wellbeing and social emotional competence of Chinese children and adolescents in the post-pandemic era: patterns, determinants, and interrelations
Source: Front Public Health. 2025 Nov 18;13:1677632. doi: 10.3389/fpubh.2025.1677632 (PMC12668964; doi:10.3389/fpubh.2025.1677632)
Supplement: Supplementary file 4 [file Data_Sheet_4.zip › Supplementary Material 4/Student_2-2_Anonymized.docx]

# Interview Notes: Student 2-2

Student 2-2

Researcher: the interviewer, the interviewer, the interviewer, the teachers

Basic Family Information:

An only child, his parents are divorced, and he lives with his mother and grandmother. However, according to the mother, the child is unaware of this. His father runs a clinic in a city in central China. He's very ambitious and busy, so he doesn't see or communicate with the child often, only seeing him two or three times a year. The mother describes him as someone who doesn't pay much attention to his studies or daily life, but provides good financial support. During the Lunar New Year, he takes the child home for the holidays.

*The mother says she used to be very busy with work and didn't pay much attention to the child, so the grandmother mostly took care of him. She recently resigned and is now a full-time caregiver. The child was observed talking to herself at the school gate and was diagnosed with depression in a city in central China. However, her mother said, "I still can't believe it, but now I have to be extra careful when talking to her at home. I plan to take her to a major hospital in a city in central China for a checkup later." She's usually quite normal, obedient and outgoing. Besides studying, she enjoys tinkering with things (crafts), so there's nothing wrong with her.*

Student 2-2's grandmother is a cancer patient. According to her mother, she may be prone to emotional outbursts. From third to sixth grade, she was primarily the child's parent. Due to her poor health, she wouldn't let the child go outside, keeping him within her sight.

Child's Relationships and Social Life

She has few friends at school and doesn't share her grades with her parents at home.

*While on a study trip with her classmates, Student 2-2 was falsely accused of stealing money. She eventually called her mother for help, proving she hadn't taken the classmate's money. Her mother later said, "After returning from that incident, she cried for a long time, even while eating." The mother also stated that she later communicated with the other student's parents, but did not apologize in person to Student 2-2.*

*Student 2-2's math grades were relatively poor, and her relationship with her math teacher was average. The math teacher once said, "Give up on her!" The mother said this was deeply hurtful and "she cried for a long time after returning home." The mother explained that every teacher has their own teaching style and expressed understanding. The parents did not contact the math teacher for further discussion. The homeroom teacher explained that because both of the math teacher's children had excellent grades, "they had never had a poor student."*

*Towards the end of the interview, the mother mentioned several times that she had asked Student 2-2 to invite classmates out, but Student 2-2 refused, or said, "You go ahead and invite them."*

Academic Performance and Performance

Overall, her grades were not very good, but she was relatively obedient. She did not serve in other class leadership roles, as the homeroom teacher stated that such roles were reserved for students who were academically strong and trustworthy.

Study Guidance

Homework Tutoring: The parents previously paid less attention to the student due to their busy work schedule.

*Regarding study habits and other requirements, the parents said, "We just want her to grow up happily, and we don't have high expectations for her grades."*

Mobile Phone and Internet Addiction: The mother said that Student 2-2 initially used her phone to surf the internet, such as Kuaishou, socializing with strangers online and sending voice messages. She was concerned, so she later confiscated her phone. She also couldn't watch TV. Because she was approaching puberty, she was worried about her browsing harmful content online.

*Student 2-2 wasn't given pocket money until sixth grade. Previously, it was always, "I'll buy you whatever you want." And after giving her pocket money, there were restrictions: "You can only buy food and drinks, not toys."*

Personality and Long-Term Development

She has a cheerful personality, is good at handicrafts, and studied sketching for a while.

*Strengths: She can cook, is considerate, and is caring. The mother said that if her child washes up first, she'll squeeze toothpaste for her and get up early to make her breakfast. If she's resting and someone in the living room talks, she'll remind them, "Keep it down! Don't disturb my mom's sleep." Student 2-2's mother wanted to take her daughter on a summer trip to a province in western China, but she said the airfare was too expensive. When it comes to money, Student 2-2 is very understanding and says her mom works hard.*

*Interviewer's self-report: After meeting the teachers, Student 2-2's family was well-off. Her mother also welcomed her warmly, offering fruit, snacks, and water. The portraits and photos on the TV stand are all of the mother and child; there are no photos of the father, which may have had some impact on the child. Furthermore, from the mother's perspective, the child is unaware of the divorce, but perhaps they already knew about it, as they don't spend the New Year together and see each other less frequently. As she told her psychologist, "I don't think Dad loves Mom." This may be one of the factors contributing to Student 2-2's low mental health score.*

*Furthermore, her grandmother's long-term "isolating" parenting may have contributed to Student 2-2's gradual introversion. She often adopts a suppressive and disapproving approach. For example, when Student 2-2 caringly tried to make breakfast for her grandmother and mother, her grandmother would refuse, saying, "I won't eat." Mom: You can just take a few symbolic bites, just to encourage her. It's clear that Mom and Grandma have some differences in their educational philosophies.*

*Furthermore, children tend to blame themselves for problems. "If only I hadn't brought so much money," Student 2-2 said when misunderstood by her peers. Furthermore, as a parent, Student 2-2 didn't promptly confront the person involved in the conflict with the teacher or classmates, which may have had a certain psychological impact on the child. "If I don't play with them, they won't play with me." There's a suspicion that there's a certain element of acting, a pretense of fitting in, but in reality, she doesn't have many close friends. This is evident from her mom's repeated refusal to invite friends out.*

*Another person who had a significant impact on Student 2-2 was likely her math teacher. Because of her poor grades, she said she was giving up, a statement that was deeply devastating for a student. The homeroom teacher stated: "Student 2-2's grades were slightly lower in the class, she wasn't very talkative, and not many children played with her." Furthermore, the homeroom teacher's words indicated that Student 2-2 wasn't particularly concerned about her. When it came to class leadership positions, she consciously chose students with better grades and didn't favor those with lower grades.*
